# Neuropathology-based approach reveals novel Alzheimer's Disease genes and highlights female-specific pathways and causal links to disrupted lipid metabolism: insights into a vicious cycle

**DOI:** 10.1186/s40478-024-01909-6

**Published:** 2025-01-04

**Authors:** Yin Jin, Apostolia Topaloudi, Sudhanshu Shekhar, Guangxin Chen, Alicia Nicole Scott, Bryce David Colon, Petros Drineas, Chris Rochet, Peristera Paschou

**Affiliations:** 1 Department of Biological Sciences, Purdue University, 915 Mitch Daniels Blvd, West Lafayette, IN USA; 2 Medicinal Chemistry and Molecular Pharmacology, Purdue University, West Lafayette, IN USA; 3 Computer Science, Purdue University, West Lafayette, IN USA

**Keywords:** Alzheimer;s disease, Neuropathology, Genomewide association study, Sex-specific analysis

## Abstract

Dementia refers to an umbrella phenotype of many different underlying pathologies with Alzheimer’s disease (AD) being the most common type. Neuropathological examination remains the gold standard for accurate AD diagnosis, however, most that we know about AD genetics is based on Genome-Wide Association Studies (GWAS) of clinically defined AD. Such studies have identified multiple AD susceptibility variants with a significant portion of the heritability unexplained and highlighting the phenotypic and genetic heterogeneity of the clinically defined entity. Furthermore, despite women’s increased susceptibility to dementia, there is a lack of sex-specific genetic studies and understanding of sex-specific background for the disorder. Here, we aim to tackle the heterogeneity of AD by specifically concentrating on neuropathological features and pursuing sex-specific analysis. We bring together 14 different genomic and neuropathology datasets (6960 individuals) and we integrate our GWAS findings with transcriptomic and phenotypic data aiming to also identify biomarkers for AD progression. We uncover novel genetic associations to AD neuropathology, including BIN1 and OPCML. Our sex-specific analysis points to a role for BIN1 specifically in women as well as novel AD loci including QRFPR and SGCZ. Post-GWAS analyses illuminate the functional and biological mechanisms underlying AD and reveal sex-specific differences. Finally, through PheWAS and Mendelian Randomization analysis, we identify causal links with AD neuropathology pointing to disrupted lipid metabolism, as well as impaired peripheral immune response and liver dysfunction as part of a vicious cycle that fuels neurodegeneration.

**Supplementary Information:**

The online version contains supplementary material available at 10.1186/s40478-024-01909-6.

## Introduction

Dementia, characterized as a persistent acquired disorder of mental processes involving memory problems, personality shifts, and impaired reasoning, ranks among the most prevalent age-related illnesses worldwide and is associated with great public health burden and costs [1]. While Alzheimer's disease (AD) is the most widespread form, other types of dementia, like vascular dementia, Lewy body dementia, and frontotemporal dementia also exist, sharing common clinical features. Thus, accurate clinical diagnosis of the specific type of dementia is challenging because multiple pathologies can give rise to similar clinical syndromes [2, 3]. AD diagnosis based on cognitive function assessments carries an approximate 24% misdiagnosis rate while neuropathological findings provide a more accurate approach [4]. This challenge also hampers genetic studies which are largely based on clinically defined AD and leave a large portion of AD heritability still unexplained [5]. Furthermore, despite higher dementia risk in women, genomic studies that investigate sex-specific genetic background are lacking. These challenges and lack of related studies leave a critical gap in our efforts to understand and tackle the clinical and sex-specific heterogeneity of dementia with a goal to drive accurate diagnosis and effective patient management.

Genome-wide Association Studies (GWAS) based on clinical AD diagnosis have led to the identification of more than 70 AD susceptibility common variants and rare genetic factors [6]. However, such studies are hampered by inclusion of pre-clinical patients in the control group and the use of a pathologically heterogeneous disease phenotype [7] which may partly explain the large portion of heritability that remains unaccounted for. Furthermore, the extent to which identified variants are risk factors for AD pathology, coexisting pathologies, or other neurobiological indices is unclear [8, 9] limiting the potential use of GWAS findings to guide drug design for AD and inform clinical trials. In fact, Farfel et al. [10], showed that many recently discovered genomic variants for AD dementia are not associated with the pathology of AD. Through neuropathological examinations, such as post-mortem brain analyses, it is possible to uncover distinctive brain abnormality patterns associated with AD, marked by the presence of neuritic plaques and tau neurofibrillary tangles. Demonstrating the power of this approach and despite using a much smaller sample than traditional GWAS, Beecham et al. [11] were able to confirm association to APOE to common AD pathologies. There is thus a need to further extend GWAS based on neuropathologically-confirmed AD.

Another factor to take into consideration in AD genomic studies is the importance of studying sex-specific differences given the observed prevalence and progression of the disease in men versus women. Women are more likely to develop AD than men, and they also exhibit more tau protein tangles in their brains, leading to faster cognitive decline compared to men [12]. Dumitrescu et al. [13] performed a sex-stratified GWAS on AD neuropathology measurements in a sample of 2,701 males and 3,275 females, the majority of whom were diagnosed with AD at autopsy. They found that, outside of the APOE region, one locus on chromosome 7 (rs34331204) showed a sex-specific association with binary NP score among males but not females, implicating a novel locus that confers male-specific protection from tau pathology. Such studies highlight the value of assessing genetic associations in a sex-specific manner.

Although post-mortem studies ensure an accurate AD diagnosis and can help tackle the heterogeneity of AD, biomarkers are needed in order to move towards risk prediction or early diagnosis and early intervention that would prevent or delay symptoms. Recent work has shown that dementia-associated pathological changes may start 20–30 years before clinical onset [14–16] and newer AD drugs are being tested on pre-symptomatic participants aiming to halt or slow down cognitive decline before substantial damage has been done to the brain. There is thus an urgent need to predict early pre-symptomatic individuals as well as aim to differentiate towards specific neuropathology that leads to cognitive decline.

Here, we tackled the phenotypic and sex-specific heterogeneity presenting a large-scale integrative analysis investigating the genomic background of neuropathologically-confirmed AD (ncAD) as well as continuous measures of Braak stage and NP score. Through neuropathology-based GWAS, sex-specific analysis and multi-omics approaches we aimed to help disentangle the heterogeneous nature of the dementia clinical phenotype to help unravel the complex pathways that lead to neurodegeneration and set the foundation for targeted therapies. Our work uncovered genes that underlie AD neuropathology and revealed insights into a vicious cycle that fuels neurodegeneration through impaired lipid metabolism, immune response, and liver dysfunction.

## Methods

### Datasets

**Fig. 1:**
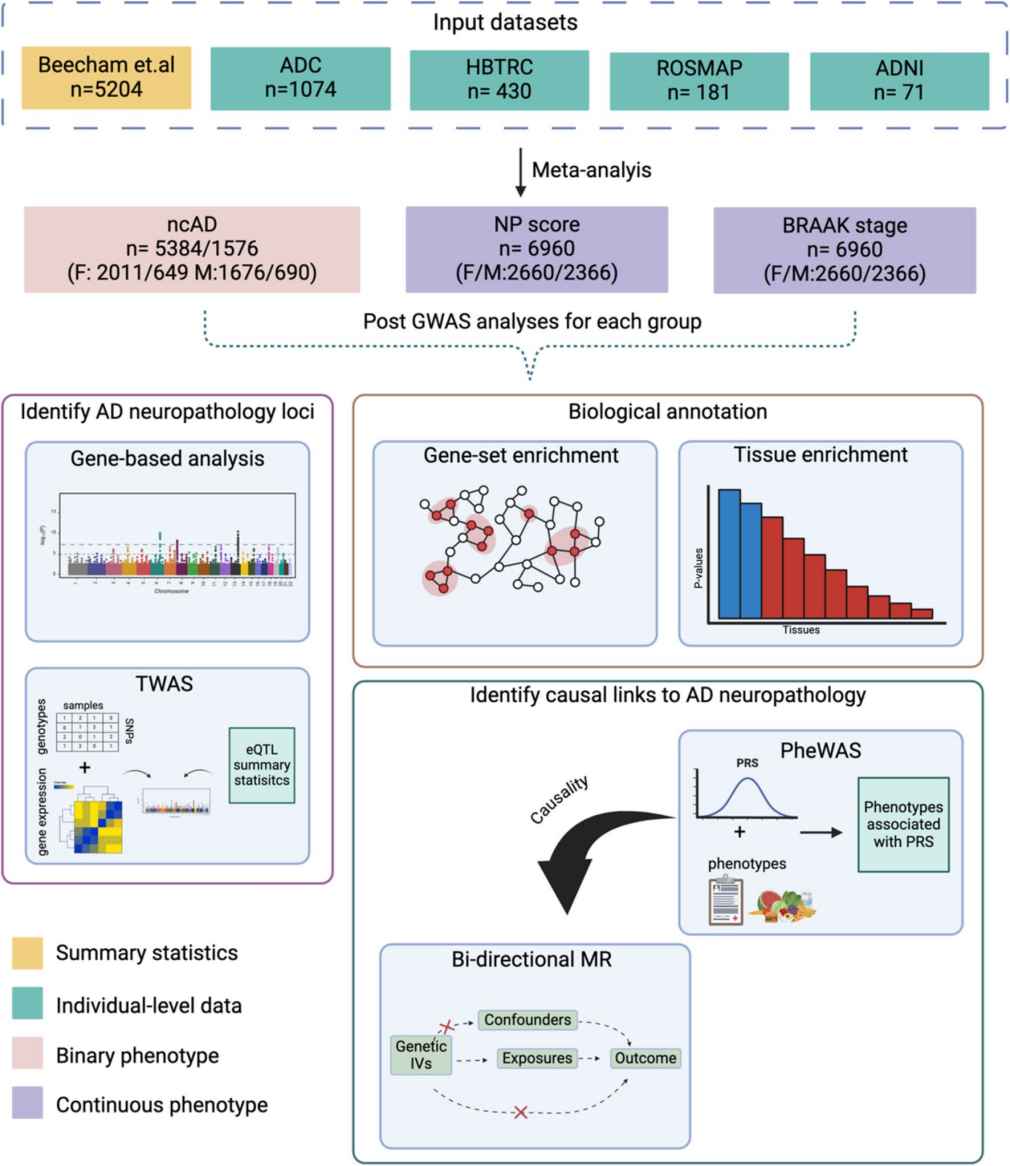
illustrates the comprehensive workflow of our study, outlining each key step from data integration to final analyses. We analyzed a total of 6,960 individuals by integrating data from 14 large-scale genetic, clinical, and neuropathology datasets, expanding upon previous Alzheimer’s Disease Genetics Consortium (ADGC) studies (Supplementary Table S1). These datasets included neuropathology assessments from the Alzheimer’s Disease Center (ADC), Religious Orders Study and Memory and Aging Project (ROSMAP), The Harvard Brain Tissue Resource Center (HBTRC), and the Alzheimer’s Disease Neuroimaging Initiative (ADNI), contributing 1756 additional samples. We conducted genome-wide association studies (GWAS) on three phenotypes: neuropathology-confirmed Alzheimer’s disease (ncAD), Braak stage, and NP score, applying stringent quality control procedures for both genotypes and variants. We performed sex-specific GWAS meta-analyses on 2660 females and 2366 males and conducted post-GWAS analyses using gene-based and gene-set approaches with FUMA and MAGMA, examining tissue specificity and gene set enrichment. Furthermore, transcriptome-wide association studies (TWAS) were performed across 13 GTEx v8 brain tissues, using the Joint-Tissue Imputation (JTI) method to identify gene regulation patterns. In addition, we conducted a PheWAS-based analysis using polygenic risk scores (PRS) to explore associations with 2248 UK Biobank phenotypes and performed Mendelian Randomization (MR) analysis to assess causal relationships between neuropathology traits and blood biomarkers from UK Biobank. Replication of significant findings was carried out using independent datasets. *ncAD: neuropathology-confirmed AD

Figure 1 illustrates the complete workflow of our analysis, from data integration to data analysis. We analyze a sample of 6,960 individuals integrating 14 large-scale genetic, clinical, and neuropathology datasets from multiple sources (Supplementary Table S1 for full details). We included summary statistics datasets as described in Beecham et al. [11] along with additional individual-level datasets, to increase the sample size compared to the prior Alzheimer's Disease Genetics Consortium (ADGC) study (full details provided in Supplementary Table S1). We expanded datasets by 1,756 samples with neuropathology assessments, including collaborative studies within ADGC, namely: (1) Alzheimer’s Disease Center (ADC) (with an increased sample size 1074), and (2) Religious Orders Study and Memory and Aging Project (ROSMAP) (with an increased sample size by 181 samples). Furthermore, we included data from The Harvard Brain Tissue Resource Center (HBTRC) study (N = 430) and the Alzheimer's Disease Neuroimaging Initiative (N = 71). The assessment of neuropathological changes on the included samples is described with more details in Supplementary Methods.

### Genomewide association studies

#### Genotyping and quality control process

The genotyping platforms that were used to assay samples in each cohort can be found in Supplementary Table S1. Standard quality control per dataset was performed as described previously [17]. Briefly, samples with call rate < 98%, heterozygosity rate > 0.2, genomic sex discrepancy with reported sex, and formation of pairs with relatedness (pi-hat) > 0.4, were excluded from the downstream analyses. Variant-level quality control was performed to exclude markers with call rate < 95%, and Hardy–Weinberg equilibrium *p*-value < 10^−6.^ To identify samples with European ancestry, Principal Component Analysis was performed with EIGENSTRAT [18] using 1000 Genomes as reference. Imputation on each dataset was performed via IMPUTE2 with 1000 Genomes as reference panel [19] using data phased by SHAPEIT [20].

#### Genetic association, meta-analysis

In each dataset association tests were performed for three phenotypes: ncAD case/control (binary), Braak stage (ordinal), and the NP score (ordinal) through PLINK [21] using the appropriate regression model (logistic for the binary phenotype and linear for the ordinal) and including the first three Principal Components (PCs) based on inspection of the data, age at death and sex as covariates. Only variants with minor allele frequency > 0.01 and info score > 0.7 (imputation quality metric) were included in the analyses. Following all quality controls in our study of AD neuropathological traits, a final meta-analysis was conducted using a total of 6960 samples (Supplementary Table S1). A fixed-effects meta-analysis was conducted using METAL, using the analytical strategy suggested by METAL authors, due to the unequal case–control ratios and study sample [22]. Variants with heterogeneity (Cochran’s Q test *p* < 0.05) and those present in less than half of the subjects were excluded.

#### Sex-specific GWAS

We performed sex-specific GWAS meta-analysis of 2660 females and 2,366 males integrating seven datasets that also had sex information available for part of their samples (Supplementary Table S2). We used the approach that was described earlier here (Methods). The sex specific GWAS for the three phenotypes was performed through PLINK including the first three PCs and age at death as covariates. For top hits, we also used GWAMA to examine heterogeneity in allelic effects between males and females, equivalent to testing genotype-sex interactions under an additive model [23].

### Post GWAS analyses

#### Gene-based and gene-set GWAS analyses

Gene-based analyses were conducted within FUMA [24] using MAGMA [25], with the 1000 Genomes dataset as a reference, and including gene density and gene size as covariates. The significance level was calculated after Bonferroni correction accounting for the tested genes. Tissue specificity analysis was performed using MAGMA with default parameters, incorporating gene expression data from GTEx v8 RNA-seq for each tissue [26], with significance set at P < 1.67 × 10^–3^ (after Bonferroni correction for 30 tissues tested) and P < 9.43 × 10^–4^ (after Bonferroni correction for 54 tissues tested). We further used MAGMA to perform gene set analyses interrogating the “GO terms” from Msigdb v7.0. P_bon_ < 0.05 was set as the significance threshold for gene-set analysis accounting for multiple-tests.

#### Transcriptome-wide association study (TWAS)

TWAS was performed using the Joint-Tissue Imputation (JTI) method [27] and with a goal of identifying genes regulated by disease-associated variants on 13 GTEx v8 brain tissues. We combined TWAS *p*-values from multiple tissues using the Aggregated Cauchy Association Test (ACAT) method [28] and performed the Bonferroni method to control for multiple tests.

### Identification of in biomarkers for AD

#### Phenome-wide association analysis (PheWAS)

In order to explore additional phenotypes associated with AD genetic risk, we performed a PRS-PheWAS analysis on UK Biobank (Supplementary Methods). As base for the PRS calculations we used the GWAS summary statistics of the three neuropathology-based phenotypes (ncAD, Braak stage, NP stage) that we performed on the full dataset and we repeated the analyses for each sex separately (N_females_ = 178,604; N_males_ = 152,237). For the PheWAS analysis we used the PHESANT tool [29] to test for PRS association on 2248 UK Biobank phenotypes (Supplementary Table S3), adjusting for the appropriate covariates (Supplementary Methods) and used FDR correction to determine the significant associations.

#### PheWAS-based on mendelian randomization (MR) analysis of blood biomarkers

We used a standard approach for two-sample MR analysis to examine the potential causal relationship between AD neuropathology traits and PheWAS significant blood assays traits in UK biobank. As exposure variables we considered SNPs with *p* < 10^−5^ in our AD neuropathological features GWAS (ncAD, Braak stage, NP stage) and the GWAS of blood assays from the UK Biobank as the outcome. Using multiple MR methods to triangulate findings provides the strongest support for causal inference. In our study, the IVW method served as the primary analysis, with the other methods (Weighted median, MR-EGGER) used for sensitivity assessments. FDR correction was applied to account for multiple testing. To validate the significant associations identified in our analyses, we used independent GWAS datasets of blood assays [30–33] as outcomes, excluding UK Biobank participants, and repeated the MR analyses. For full details, refer to the Supplementary Methods, and see Supplementary Table 4 for information on the datasets.

## Results

### Genome-wide association studies of AD neuropathology

**Fig. 2:**
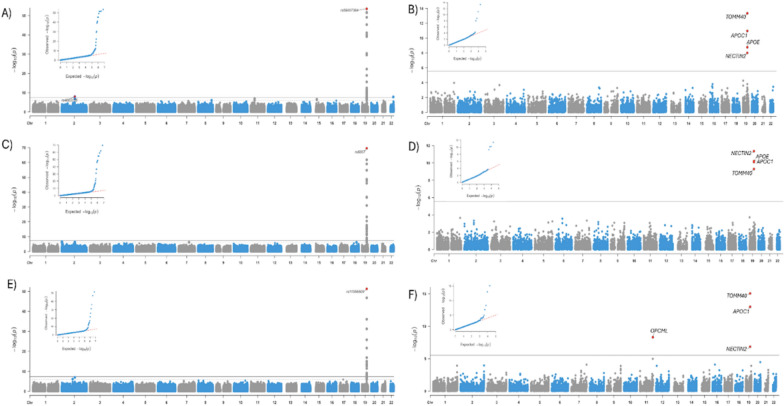
Manhattan and QQ plots of SNP-based and Gene-Based genome-wide association results of neuropathological features of AD (n = 6960). Dotted red lines represent the threshold for genome-wide significance (P < 5 × 10^−8^) and Bonferroni correction for the gene-based analyses. **A** Neuropathologically-confirmed AD case–control GWAS. **B** Gene-based analysis for neuropathologically confirmed AD case–control sample. **C** Braak stage GWAS. **D** Gene-based analysis for Braak stage. **E** NP score GWAS. **F** Gene-based analysis for NP score

First, we integrated 14 large-scale genetic, clinical, and neuropathology datasets from multiple sources and conducted GWAS meta-analyses for three neuropathology-based phenotypes: ncAD (neuropathology-confirmed AD) on a total of 5384 cases and 1576 controls, Braak stage, and NP stage on 6960 individuals (see Methods as well as Supplementary Materials, Supplementary Table S1). We identified two genomewide significant loci associated to AD neuropathology (see Table 1, Supplementary Fig. 2 for regional plots and Supplementary Fig. 3 for forest plots). The top and only locus shared by the three GWAS that we performed was 19q13.32 near the APOE region (spanning TOMM40, APOE, NECTIN2 and APOC1) (Table 1, Fig. 2). The second genome-wide significant locus shared by case–control ncAD GWAS was on chromosome 2q14 on the BIN1 gene (Table 1, Fig. 2, Supplementary Figure S2 for regional plot).

**Table 1:** Genome-wide association study of neuropathology-based AD traits

Locus	CHR:POS	Top SNP ID	AD traits	A1	P	Effect	Nearest Gene Position (distance)	MAF
2q14.3	chr2:127,133,851	rs4663105	ncAD	C	7.94 × 10^–9^	− 5.77	BIN1^b^/intergenic (26,495)	0.4
	chr19:44,893,408	rs59007384	ncAD	T	3.9 × 10^−31^	15.52	APOE region^a^/intronic	0.30
19q13.32	chr19:44,888,997	rs6857	Braak stage	T	2.7 × 10^−70^	17.73	APOE region^a^/intronic	0.25
	chr19:44,892,887	rs11556505	NP score	T	5.2 × 10^−52^	15.18	APOE region^a^/intronic	0.22

The total sample for the ncAD case–control GWAS was 5384 cases and 1576 controls on 6,394,125 SNPs. The total sample for Braak stage and NP score GWASs was 6960 individuals on 6,542,713 and 6,475,755 SNPs respectively. CHR chromosome; SNP, single-nucleotide polymorphism; A1, effect allele; MAF: minor allele frequency; Effect: Z score effect of A1 allele; Significant genome-wide association P < 5 × 10^−8^ a: Genes have been identified as genome-wide significant in both previous clinical AD GWAS and neuropathological AD GWAS. b: Genes have been identified as genome-wide significant in previous clinical AD GWAS

ncAD: neuropathology-confirmed AD

In the gene-based analyses, the TOMM40, NECTIN2, and APOC1 genes were significantly associated with all three studied neuropathology-based AD-related phenotypes (Fig. 2B, D, F). The APOE gene was also significant in the ncAD and Braak stage meta-analysis (Fig. 2B, D), while one novel gene, OPCML, outside of the 19q13.32 region was significant in the NP score GWAS (Fig. 2F).

### Sex-specific genome-wide association studies of AD neuropathology

**Fig. 3:**
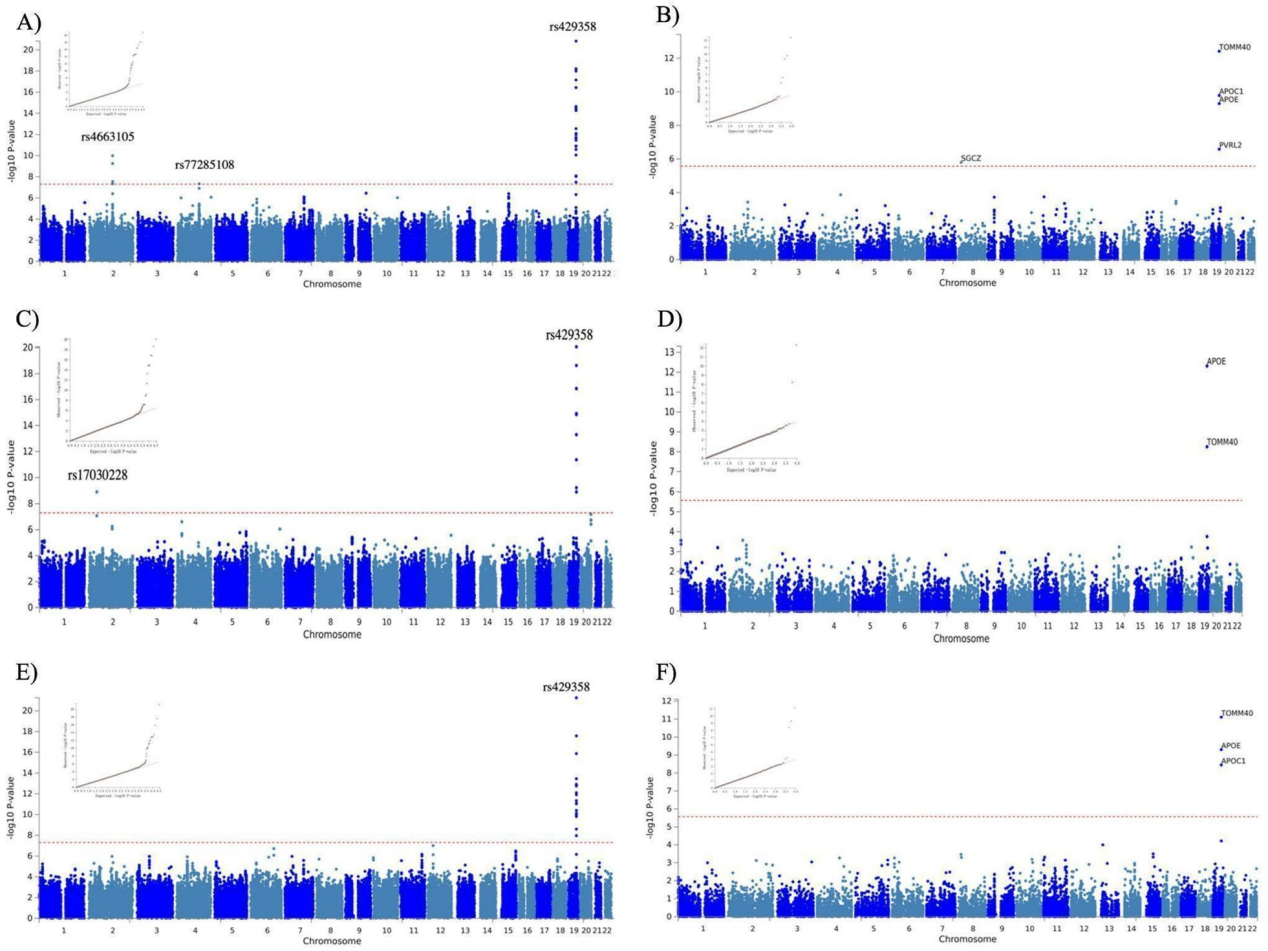
Manhattan and QQ plots of SNP-based and Gene-Based female-specific GWAS results of neuropathological features of AD (n = 2660). Dotted red lines represent the threshold for genomewide significance (P < 5 × 10^−8^) and Bonferroni correction for the gene-based analyses. **A** ncAD female-specific GWAS. **B** Gene-based analysis for ncAD female-specific case–control sample. **C** Braak stage female-specific GWAS. **D** Gene-based analysis for Braak stage in females. **E** NP score female-specific GWAS. **F** Gene-based analysis for NP score in females

Next, aiming to investigate sex-specific genetic associations with AD neuropathology traits, we performed sex-stratified GWAS after merging seven datasets with available sex information (Supplementary Table S2 and Fig. S6 forest plots). This resulted in a total of 2366 males and 2660 females. As expected, for both sexes, the top locus in all three AD neuropathology phenotypes that we studied (ncAD, Braak stage, NP score) was 19q13.32 with the top SNPs located in the APOE region (Table 2, Fig. 3, Supplementary Figure S1). No additional significant loci were identified in the male specific GWAS for any of the three phenotypes (Table 2). However, SNPs at two additional loci exceeded the genomewide significance level in the female-specific GWAS for Braak stage and ncAD: 2q14 close to the BIN1 gene and 4q27 close to QRFPR were significantly associated with ncAD in females (top SNPs: (2q14) rs4663105, *p*-value = 1.01 × 10^–10^; (4q27) rs77285108, *p*-value = 1.23 × 10^–9^) (Fig. 3C, E, Supplementary Figure S5A-B for regional plots). Furthermore, rs17030228 close to the LOC102723854 was significant in the female-specific Braak stage GWAS (top SNP:rs17030228, *p*-value = 8.5 × 10^–8^) (Table 2, Fig. 3C and Supplementary Figure S5C for regional plot). The sex heterogeneity test revealed that the effects of rs4663105, rs17030228 and rs77285108 were significantly different between males and females (all sex heterogeneity *p*-value < 0.05 and Supplementary Table S5). In sex-specific gene-based analysis, several genes in the APOE region (APOC1 TOMM40, PVRL2) were significant for all three AD neuropathology traits for both sexes, while the SGCZ gene was significant in female specific ncAD only (Fig. 3B, D, F, Supplementary Figure S1B, S1D, S1F).

**Table 2:** Sex-specific genome-wide association study of neuropathology-based AD traits

Locus	CHR(POS)	SNP ID	AD traits	A1	Female only *p*-value	Female only Effect size	Male only *p*-value	Male only Effect size	Nearest Gene Position (dis)	MAF
2q14	chr2:127,133,851	rs4663105	ncAD	C	1.01 × 10^–10^	− 6.47	7.5 × 10^–4^	− 3.37	BIN1b/intergenic (26,495)	0.4
2q	chr2:42,953,187	rs17030228	Braak stage	A	1.23 × 10^–9^	− 6.08	0.49	− 0.17	AC016735.1 lncRNAsc/intergenic (48,167)	0.012
4q27	chr4:121,361,613	rs77285108	ncAD	G	4.67 × 10^–8^	5.46	0.52	− 0.65	QRFPRc/intronic	0.15
19q13.32	chr19:44,908,684	rs429358	ncAD	C	1.50 × 10^–21^	− 9.54	8.65 × 10^–32^	− 11.73	APOE regiona/exonic	0.16
	chr19:44,908,684	rs429358	Braak stage	C	8.50 × 10^–21^	− 9.35	1.96 × 10^–34^	− 12.24	APOE regiona/exonic	0.16
	chr19:44,908,684	rs429358	NP score	C	5.65 × 10^–22^	− 9.64	1.08 × 10^–27^	− 10.91	APOE regiona/exonic	0.16

The total sample for the ncAD female case–control GWAS was 2660 (2011 case + 649 control) and male was 2366 (1676 case + 690 control) on 7,045,108 and 7,075,025 SNPs respectively. The total sample for female Braak stage GWASs and female NP score GWASs was 2660 on 7,083,498 and 61,890,055 SNPs respectively. The total sample for male Braak stage GWASs and male NP score GWASs was 2366 individuals on 7,153,692 and 6,475,237. CHR chromosome; SNP, single-nucleotide polymorphism; A1, effect allele; MAF: minor allele frequency; Effect: Z score effect of A1 allele; Significant genome-wide association P < 5 × 10^−8^.a: Genes have been identified as genome-wide significant in both previous clinical AD GWAS and neuropathological AD GWAS. b: Genes have been identified as genome-wide significant in previous clinical AD GWAS. c: Genes have not been identified as genome-wide significant in previous studies

*ncAD* neuropathology-confirmed AD

### Post-GWAS analysis for AD neuropathology

**Fig. 4:**
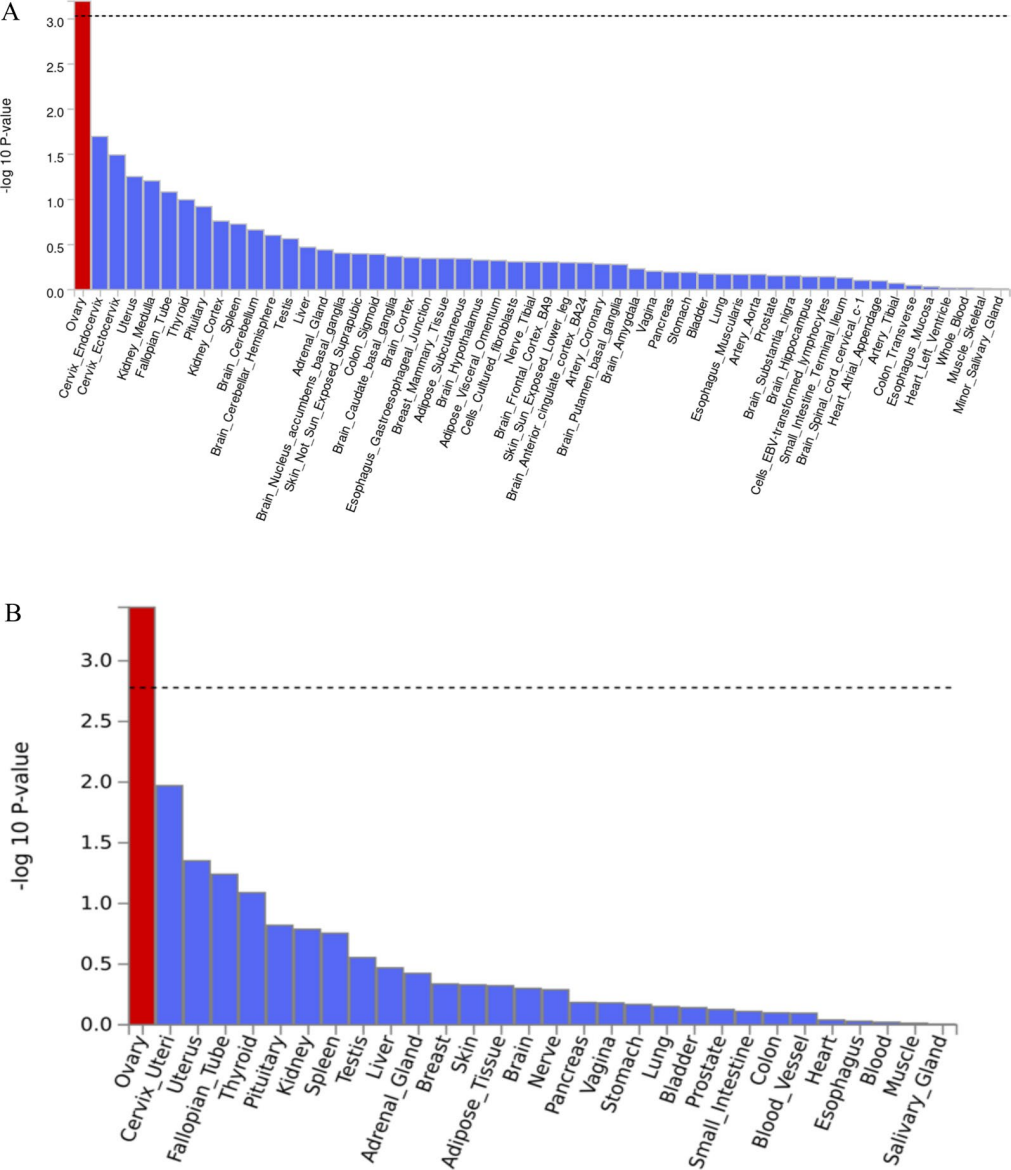
Post-GWAS analysis results for Tissue enrichment analysis. Tissue enrichment analysis for NP score GWAS results in females. The analysis was performed in MAGMA using GTEx v8 RNA-seq data 54 and 30 general tissue types. With red are shown the significant results after multiple testing corrections. **A** MAGMA tissue expression analysis using gene expression per tissue based on GTEx RNA-seq data for 54 specific tissue types. **B** MAGMA tissue expression analysis using gene expression per tissue based on GTEx RNA-seq data for 30 specific tissue types

To identify tissue specificity of our AD neuropathology GWAS findings, we performed tissue enrichment analysis by MAGMA (Supplementary Figure S4 and S7). Notably, a significant association was observed between NP score in females and genes expressed in ovary tissue across both the 54- and 30-tissue enrichment analyses (Fig. 4). In our gene-set analysis, we found several significant enrichments: In males, the NP score showed enrichment for “positive regulation of mitochondrial calcium ion concentration” (P_bon_ = 3.6 × 10^–2^), while for male ncAD, "histone pre mRNA 3' end processing complex" (P_bon_ = 3.5 × 10^–2^) was significant. In females, the NP score revealed "regulation of luteinizing hormone secretion" (P_bon_ = 1.2 × 10^–5^) and "positive regulation of gonadotropin secretion" (P_bon_ = 1.7 × 10^–3^). Additionally, in females, "positive regulation of receptor catabolic process" (P_bon_ = 2.6 × 10^–2^) and "fibroblast migration" (P_bon_ = 2.2 × 10^–2^) were enriched for Braak stage and case–control analysis, respectively. (Supplementary Table S6).

**Fig. 5:**
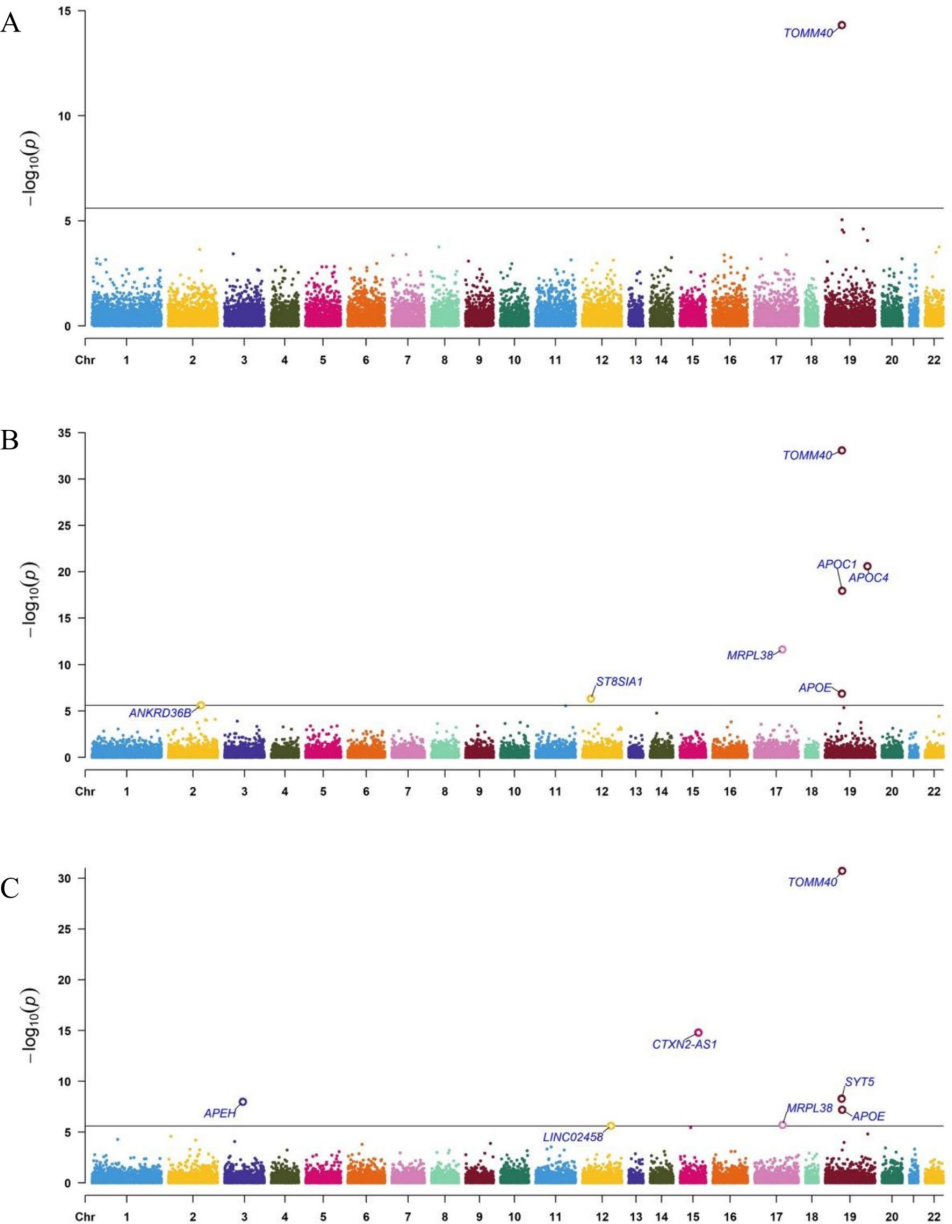
Transcriptome-wide association study (TWAS) for AD neuropathological features. The x-axis of Manhattan plot represents the genomic position of the corresponding gene, and the y-axis of Manhattan plot represents -log10-transformed association combined *P*-value using ACAT. Each dot represents the association for one specific gene. The line shows combined P value 9.71 × 10^–6^. **A** TWAS for neuropathologically confirmed AD case–control sample. **B** TWAS for Braak stage. **C** TWAS for NP scor

To identify candidate genes whose genetically regulated expression is associated with neuropathological features of AD, we conducted TWAS (see Table 3 and Fig. 5). This analysis identified 11 protein-coding and two significant long non-coding RNA gene hits whose transcript expression was significantly associated with neuropathological features of AD. Notably, TWAS identified six novel loci including genes (ST8SIA1 *p*-value = 4.69 × 10^–7^; ANKRD36B *p*-value = 3.38 × 10^–6^; MRPL38 *p*-value = 2.34 × 10^–12^ and 1.97 × 10^–6^, APEH *p*-value = 1.03 × 10^–8^; CTXN2-AS1 p-value = 1.60 × 10^–15^; LINC02458 *p*-value = 2.41 × 10^–6^) and one novel gene in the APOE region (SYT5 *p*-value = 5.29 × 10^–9^). These genes have not been implicated in previous AD-related GWAS or TWAS and are novel findings of this study.

**Table 3:** Transcriptome-wide association study of neuropathology-based AD traits

GWAS summary	Gene	Region	ACAT P	Leading tissues
Braak Stage	ST8SIA1 ^c^	12p12.1	4.69 × 10^–7^	Brain_Cerebellar_Hemisphere, Brain_Cerebellum
	APOC1 ^a^	19q13.32	1.18 × 10^–18^	Nucleus_accumbens_basal_ganglia
	APOE ^a^	19q13.32	1.37 × 10^–7^	Brain_Caudate_basal_ganglia,
	TOMM40 ^a^	19q12.32	8.33 × 10^–34^	Pituitary
	ANKRD36B ^c^	2q11.2	3.38 × 10^–6^	Brain_Cortex Brain_Hippocampus Nucleus_accumbens_basal_ganglia Brain_Spinal_cord_cervical_c-1 Brain_Substantia_nigra
	MRPL38 ^c^	17q25.1	2.34 × 10^–12^	Brain_Hypothalamus
	APOC4 ^a^	19q13.31	2.67 × 10^–21^	Brain_Caudate_basal_ganglia, Brain_Hypothalamus Nucleus_accumbens_basal_ganglia
ncAD	TOMM40 ^a^	19q13.32	5.43 × 10^–16^	Pituitary
NP score	SYT5 ^c^	19q13.42	5.29 × 10^–9^	Brain_Cerebellar_Hemisphere
	APOE ^a^	19q13.32	6.51 × 10^–8^	Brain_Caudate_basal_ganglia
	TOMM40^a^	19q13.32	1.88 × 10^–31^	Pituitary
	APEH^c^	3p21.31	1.03 × 10^–8^	Brain_Cerebellar_Hemisphere
	MRPL38^c^	17q25.1	1.97 × 10^–6^	Brain_Hypothalamus
	LINC02458 ^c^	12q21.33	2.41 × 10^–6^	Brain_Frontal_Cortex_BA9
	CTXN2-AS1 ^c^	15q21.1	1.60 × 10^–15^	Pituitary

Transcriptome-wide association study (TWAS) identifies 11 unique and 7 novel genes significantly associated with neuropathological features of AD in GTEx Brain tissues. GWAS: Genome-wide association study; TWAS: Transcriptome-wide association study; ACAT P: Aggregated Cauchy Association Test based combined TWAS *p*-value Bonferroni correction thresholds are *p*-value < 2.48 × 10–6 based on 20,205, 20,198, and 20,207 tests in Braak stage, ncAD case–control, and NP score combined JTI-ACAT TWAS analysis respectively. a: Genes have been identified as significant in previous clinical or neuropathology-based AD GWAS. b: Genes have been identified as significant in previous AD-related TWAS alone, c: Genes have not been identified as significant in previous AD GWAS or TWAS studies. * ncAD: neuropathology-confirmed AD

### Investigating potentially causal links with AD neuropathology

#### PheWAS and PheWAS-based on MR

**Fig. 6:**
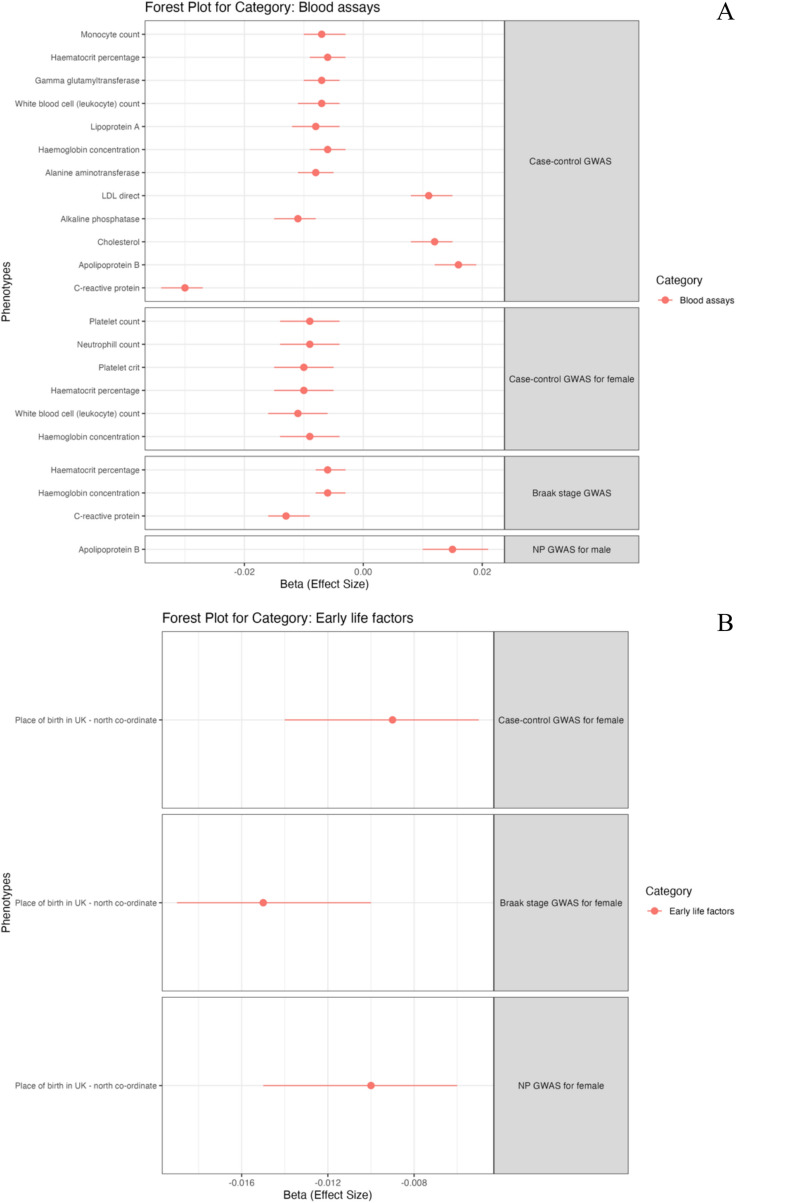

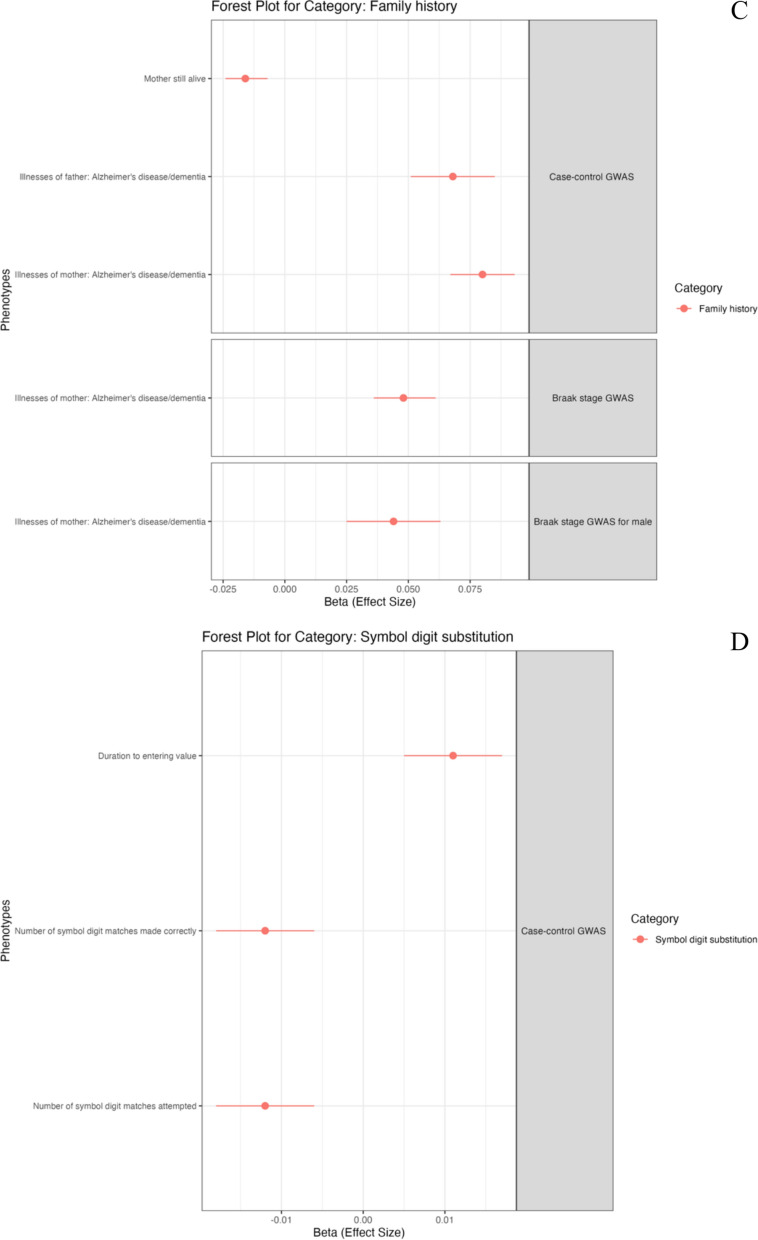

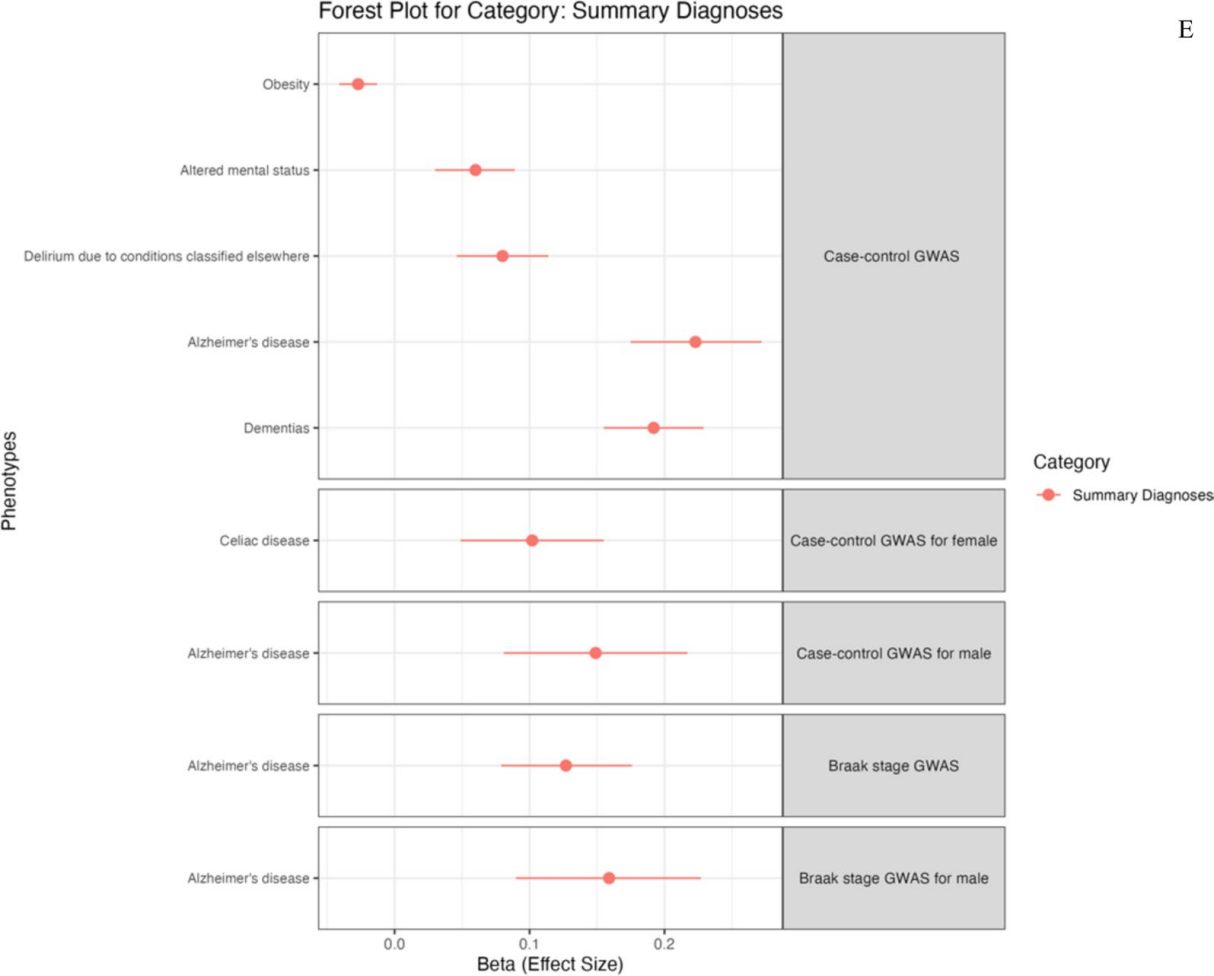
Phenome-wide association analysis (PheWAS) for PRS of neuropathology-based AD GWAS. Forest plot showing phenotypes significantly associated with TS PRS, grouped by categories The x-axis shows the (Beta) effect size for each phenotype estimated by PheWAS. **A** Forest plot for blood assay. **B** Forest plot for early life factors **C** Forest plot for family history. **D** Forest plot for cognitive function of symbol digit substitution. **E** Forest plot for ICD10 diagnosis summary

We continued to perform PheWAS to identify associations between genetic variation and phenotypic variation in European populations from the UK Biobank dataset. Our PRS-PheWAS results are presented in Fig. 6 and Supplementary Table 7. The ncAD PRS showed significant associations with 36 traits while we found 10 associations with Braak stage PRS. Interestingly, in the blood biomarkers category ncAD was associated with increased lipid metabolism (apolipoprotein B, LDL direct and cholesterol) and decreased transferase (alkaline phosphatase, alanine aminotransferase and gamma glutamyltransferase). It was also negatively associated with C-reactive protein (CRP) and blood cells counts measurements. Braak stage PRS was also associated with elevated lipid metabolism and decreased CRP. Another interesting result was the inverse relationship between ncAD PRS and obesity. In the sex-specific PheWAS analysis, the majority of associations were linked to blood biomarkers specifically in females. Among disease diagnoses, female ncAD PRS was linked to celiac disease, while male ncAD and Braak stage PRSs were associated with AD (more details in supplementary table 7 and supplementary results).

We proceeded to further explore the PheWAS blood assay associations investigating potential causality. To do this, we conducted a bi-directional MR analysis (Table 4). We identified a causal relationship where neuropathology, particularly in ncAD, led to changes in blood assay traits. Specifically, there were positive causal associations between ncAD and lipid metabolism markers, including cholesterol, LDL direct, and apolipoprotein B. In contrast, several negative causal relationships were found, with ncAD linked to decreased levels of CRP, alkaline phosphatase, alanine aminotransferase, and blood cell count measurements. Braak stage also showed a negative causal link with CRP and a positive causal relationship with most lipid metabolism markers. Additionally, we successfully replicated the causal associations of ncAD with cholesterol, CRP, platelet crit, LDL direct, and red cell count in independent blood assay datasets. We also replicated the causal effect of Braak stage In the reverse direction, LDL was found to directly contribute to Braak stage.on lipid metabolism markers although this could not be replicated when using independent GWAS. Sex-specific analysis did not reveal significant results. Full details of methods and results are provided in Supplementary text and Table S8.

**Table 4:** Mendelian randomization analysis and replication results

Exposure	Outcome	MR analysis					Replication			
		N SNPs	Beta	SE	P value	FDR	N SNPs	Beta	SE	*p* value
Case–control GWAS	Alanine aminotransferase	20	− 0.024	0.012	0.041	0.057				
	Alkaline phosphatase	20	− 0.030	0.012	0.015	0.032*^a^				
	Apolipoprotein A	20	− 0.068	0.021	0.012	0.032*	20	− 0.003	0.022	0.895
	Apolipoprotein B	19	0.126	0.054	0.019	0.032*	20	0.064	0.091	0.483
	Cholesterol	20	0.082	0.035	0.019	0.032*	20	0.096	0.035	0.006*
	C-reactive protein	20	− 0.192	0.064	0.003	0.032*	20	− 0.181	0.077	0.018*
	Gamma glutamyltransferase	20	− 0.008	0.008	0.341	0.38				
	Haematocrit percentage	20	− 0.008	0.008	0.341	0.39				
	Haemoglobin concentration	20	− 0.001	0.004	0.803	0.803				
	HDL cholesterol	20	− 0.028	0.014	0.043	0.050				
	LDL direct	20	0.095	0.041	0.020	0.032*	20	0.093	0.040	0.020*
	Monocyte count	20	− 0.006	0.008	0.475	0.50				
	Platelet count	20	− 0.020	0.008	0.014	0.032*	20	− 0.041	0.025	0.094
	Platelet crit	20	− 0.025	0.010	0.014	0.032*	20	− 0.054	0.018	0.003*
	Red blood cell (erythrocyte) count	20	− 0.014	0.005	0.007	0.032*	20	− 0.004	0.019	0.845
	Red blood cell (erythrocyte) distribution width	20	− 0.037	0.013	0.004	0.032*	20	− 0.041	0.015	0.007*
Braak stage GWAS	Apolipoprotein A	25	− 0.061	0.024	0.012	0.020*	26	− 0.063	0.022	0.004*
	Apolipoprotein B	24	0.005	0.005	0.324	0.320				
	Cholesterol	24	0.007	0.006	0.256	0.320				
	C-reactive protein	25	− 0.176	0.058	0.002	0.008*	25	− 0.087	0.069	0.210
	LDL direct	26	26.000	0.126	0.003	0.008*	26	0.133	0.042	0.002*

The table presents the results of a Mendelian Randomization (MR) analysis and subsequent replication studies investigating the causal relationships between genetic risk factors (exposures) from ncAD and Braak stage GWAS and various blood biomarkers (outcomes) identified from previous PheWAS analysis. Exposure: Either ncAD GWAS or Braak stage GWAS, representing the genetic risk factors being tested. Outcome: The specific blood biomarkers identified through PheWAS analysis, which are the traits being evaluated for causal relationships with the genetic exposures. Replication: Results from replication studies, including the number of SNPs (N SNPs), beta coefficient, standard error (SE), and *p*-value for select outcomes that were re-tested to confirm consistency in the results. This table focuses on the primary inverse variance-weighted (IVW) analysis, with additional methods and sensitivity tests available in Supplementary Table 8. An asterisk (*) indicates a significant cutoff; The letter "a" indicates that the replicate dataset is not available

## Discussion

We performed a large-scale AD-neuropathology-based GWAS, revealing sex-specific AD pathways and novel AD loci that had not been previously found in clinical AD GWAS. The BIN1 gene, which has been previously identified as associated with clinical AD [34] and had not been associated in an original AD neuropathology GWAS [11] is highlighted by our analysis. Importantly, we show, for the first time, that BIN1 is specifically associated with AD neuropathology in females and not in males. BIN1 plays a prominent role in regulating endocytosis and synaptic vesicle trafficking, and it is implicated in the generation of amyloid beta, mediation tau pathology and the propagation of Tau [35–38]. Recent sex-specific clinical GWAS and APOEε4 status GWAS for AD, also identified BIN1 as having a female-specific association [39, 40]. Another investigation, estimating hazard ratios, also showed that BIN1 contributes to a higher risk in females compared to males [41]. Moreover, GTEx RNAseq analysis has previously underscored the sex-heterogeneous effect of BIN1 in brain tissue [42]. These findings suggest that the effects of BIN1 may be sex-dependent, particularly in the context of AD neuropathology.

The different factors that operate towards progression to AD in men and women and the increased risk in women are multifactorial and both sex hormones and sex chromosomes have been implicated [43]. Our sex-specific analysis of AD neuropathology supports an important role for sex hormones. We found high expression of our top GWAS hits in the ovary and our gene-set analysis connected NP score to pathways that are related to the secretion of luteinizing hormone (LH) and gonadotropins in females. LH is a component of the Hypothalamus-Pituitary-Gonads axis and becomes dysregulated during aging, particularly in menopause. Although the potential role of estrogen [44, 45] in AD has received a lot of attention, emerging data [46] suggest an important role for luteinizing hormone in the function of the central nervous system and post-menopausal women have up to tenfold more LH than men [47, 48]. Mounting evidence suggests that such hormone changes during perimenopause contribute to female vulnerability to AD and our work here highlights this mechanism [49, 50].

Besides BIN1, which we discussed earlier here, our sex-specific analysis identified novel AD genes with female-specific association to AD neuropathology. These include genes QRPFR, SGCZ, and the long non-coding RNA (lncRNA) AC016735.1. QRPFR (GPR103) is highly expressed in the brain and acts as a receptor for the orexigenic neuropeptide, influencing the regulation of feeding behavior and circadian rhythms [51, 52]. Interestingly, intra-hippocampal administration of orexin has been shown to mitigate learning and memory impairment, highlighting its potential therapeutic role in AD [53]. Furthermore, disrupted circadian rhythms have been previously linked to AD development, further supporting a potential role of QRPFR in AD pathology [54, 55]. Additionally, QRPFR exhibits a neuroprotective effect, and its expression is reduced in AD due to amyloid-beta and tau pathology. SGCZ, another novel female-specific AD-neuropathology gene that we identified, has been shown to play a role in forming the sarcoglycan complex and exhibits gender-biased expression levels in the brain, as observed in animal models [56]. Mutations in sarcoglycanopathy can lead to protein misfolding and aggregation, which could potentially be connected to the development of AD [57]. A single-cell analysis study revealed elevated expression of SGCZ in a subset of oligodendrocytes when comparing individuals with AD to those without the condition [58]. We also found AD neuropathology gene (OPCML: Opioid-Binding Protein/Cell Adhesion Molecule) that had not been previously identified in neuropathology GWAS or the largest clinical AD GWAS. OPCML belongs to the immunoglobulin protein superfamily and contributes to synaptogenesis in the brain [59]. It has been implicated in AD based on previous GWAS studies in the Dutch population [60].

Through TWAS we also identified seven additional novel genes as regulated by ncAD GWAS variants. (ST8SIA1, ANKRD36B, MRPL38, APEH and CTXN2-AS1, SYT5, and LINC02458). Intriguingly, ST8SIA1 encodes GD3 synthase which is involved in regulating amyloid-beta plaque load [61]. APEH has been linked to endogenous beta-amyloid levels and is also associated with decreased red blood cell counts in AD patients [62, 63]. Additionally, the two lncRNA genes that are implicated by our analysis, LINC02458 and CTXN2-AS1 have been found to influence processes like amyloid beta aggregation, tau hyperphosphorylation, and the interaction of key enzymes in AD by acting as a decoy or scaffold [64]. Further biological experimentation is warranted to provide additional support for the implication of these genes in AD pathology.

Intriguingly, our MR analysis provided evidence that AD-related neuropathology leads to disruption of lipid metabolism, and increased levels of cholesterol, LDL, and apolipoprotein B. Previously, high cholesterol, and LDL have been implicated as risk factors for AD [65] and here we show evidence also for a reverse relationship, pointing to a vicious cycle that fuels neurodegeneration. We also showed that AD neuropathology can cause a decrease in CRP levels, as well as lower levels of liver enzymes, pointing to liver dysfunction, and reduced peripheral immune response that could further aggravate neurodegeneration. Lower CRP levels, an inflammatory biomarker, have been associated with a higher risk of AD in a large population study [66]. Although sex-specific MR did not reveal significant results of causality, in PheWAS we observed significant associations between blood assays, sociodemographic traits, and neuropathological feature PRS, with notable sex differences. In females, we identified negative associations between ncAD PRS and white cell, platelet, and red cell counts, aligning with previous studies that reported decreased peripheral blood cells in AD [67, 68].

## Conclusions

In summary, we used a multi-omics approach centered on GWAS to explore the genetic basis of AD neuropathology, with a focus on sex-specific mechanisms and investigating potential causal links for AD. Our GWAS identified strong associations with AD neuropathology, including novel loci such as BIN1, which was highlighted as having a female-specific association. Further analyses through tissue-specificity, gene-set, TWAS, and MR provided additional insights into the role of these genetic variants, implicating key biological pathways. Notably, our findings suggest that sex-specific factors, such as hormone regulation, may play a critical role in the progression of AD, particularly in females. MR analysis further integrated GWAS findings with clinical data from PheWAS, identifying an intriguing bi-directional link to lipid metabolism. The combined approach of GWAS, TWAS, MR, and post-GWAS analyses helped us provide novel insights into the progression of AD neuropathology. Further research is warranted to further validate and understand the suggested interplay between lipid dysregulation, liver function, and inflammation in AD which may offer new therapeutic opportunities to break the cycle and slow down disease progression.

## Supplementary Information

Additional file 1.

Additional file 2.

## Data Availability

The ADGC cohorts’ data is available at niagads (https://www.niagads.org/), while the ADNI dataset can be accessed from https://adni.loni.usc.edu/. Other data from Religious Orders Study and Memory and Aging Project, Mayo Clinic Alzheimer's Disease Research Center, and Harvard Brain Tissue Resource Center are accessible on https://www.synapse.org.

## Abbreviations

ncAD: Neuropathologically-confirmed AD
AD: Alzheimer’s disease AD
GWAS: Genome-wide association studies
ADGC: Alzheimer’s disease genetics consortium
ADC: Alzheimer’s disease center
ROSMAP: Religious orders study and memory and aging project
HBTRC: Harvard Brain Tissue Resource Center
PCs: Principal components
TWAS: Transcriptome-wide association study
JTI: Joint-tissue imputation
ACAT: Aggregated Cauchy association test
PheWAS: Phenome-wide association analysis
MR: Mendelian randomization
CRP: C-reactive protein
LH: Luteinizing hormone
lncRNA: Long non-coding RNA
OPCML: Opioid-Binding Protein/Cell Adhesion Molecule
