# Supplementary material for: Neuropathology-based approach reveals novel Alzheimer's Disease genes and highlights female-specific pathways and causal links to disrupted lipid metabolism: insights into a vicious cycle
Source: Acta Neuropathol Commun. 2025 Jan 4;13:1. doi: 10.1186/s40478-024-01909-6 (PMC11699708; doi:10.1186/s40478-024-01909-6)
Supplement: Supplementary file 1 — Additional file 1. [file 40478_2024_1909_MOESM1_ESM.docx]

**Supplementary Appendix**

**Supplement to:**

[Description of datasets 2](#_Toc179640142)

[Supplementary Methods 3](#_Toc179640143)

[Supplementary Results 5](#_Toc179640144)

[Figure S1: Manhattan and QQ plots of SNP-based and Gene-Based genome-wide association results of neuropathological features of AD in males (n=2366). 8](#_Toc179640145)

[Figure S2 Regional association plots for locus significantly associated with neuropathological features of AD. 9](#_Toc179640146)

[Figure S3 Forest plots for locus significantly associated with neuropathological features of AD. 11](#_Toc179640147)

[Figure S4: Tissue enrichment analysis for neuropathologically confirmed AD GWAS results. 12](#_Toc179640148)

[Figure S5 Regional association plots for locus significantly associated with neuropathological features of AD in females. 14](#_Toc179640149)

[Figure S6 Forest plots for locus significantly associated with sex specific neuropathological features of AD. 21](#_Toc179640150)

[Figure S7: Tissue enrichment analysis for neuropathologically confirmed AD sex specific GWAS results. 22](#_Toc179640151)

[Reference 24](#_Toc179640152)

#

# Description of datasets

ADGC spans 12 cohorts: the Adult Changes in Thought study (ACT), Alzheimer’s Disease Center (ADC), the Mayo Clinic Alzheimer's Disease Research Center (MAYO), the University of Miami Brain Endowment Bank (MBB), the National Institute on Aging Late-Onset Alzheimer's Disease Family Study (NIA-LOAD), the Oregon Health & Science University Alzheimer's Disease Center (OHSU), the Religious Orders Study and Memory and Aging Project (ROSMAP), the Translational Genomics Research Institute (TGEN), the University of Pittsburgh Alzheimer's Disease Research Center (UPI), the University of Miami Hussman Institute for Human Genomics (UM), the Vanderbilt University Center for Human Genetics Research (VU), and the Mount Sinai School of Medicine (MSSM) cohort. Here, we put together a sample of 6,960 individuals integrating 14 large-scale genetic, clinical, and neuropathology datasets from multiple sources (Supplementary Materials, Supplementary Table S1). We included summary statistics datasets as described in Beecham et al. along with additional individual-level datasets, to increase the sample size compared to the prior Alzheimer's Disease Genetics Consortium (ADGC) study (full details provided in Supplementary Table S1). The expanded datasets included collaborative studies within ADGC, namely: 1) Alzheimer’s Disease Center (ADC) (with an increased sample size 1,074), and 2) Religious Orders Study and Memory and Aging Project (ROSMAP) (with an increased sample size 181). Furthermore, we included data from The Harvard Brain Tissue Resource Center (HBTRC) study (N=430) and the Alzheimer's Disease Neuroimaging Initiative (N=71).

# Supplementary Methods

***Neuropathological changes assessment***

Samples were assessed for neuropathological changes using the guidelines recommended by the National Institute on Aging and the Ronald and Nancy Reagan Institute of the Alzheimer's Association. Criteria for (neuropathology-confirmed) ncAD cases and controls were based on CERAD scores for NPs and Braak staging scores for NFTs [1][2]. For ncAD, a NP score of moderate/frequent and Braak stage III–VI were used, while for controls, a NP score of sparse and Braak stage less than II, or a NP score of none and Braak stage less than IV were used.

***Post-GWAS analyses***

*Phenome-Wide Association Analysis (PheWAS) and PheWAS-based on MR*

In order to explore additional phenotypes associated with AD genetic risk, we performed a PheWAS. We initiated our analysis by computing PRS scores via the PRScs software[3] for all UK Biobank participants of European ancestry (N=330,841). The UK Biobank quality control and selection of individuals has been previously described [4]. As base for the PRS calculations we used the GWAS summary statistics of the three neuropathology-based phenotypes that we performed on the full dataset (ncAD, Braak stage, NP stage). Additionally, we repeated the analyses for each sex separately; using the sex-specific neuropathology-based GWAS as base and UK Biobank samples as target (N_females_=178604; N_males_=152237), we calculated the sex-specific PRS for each of the three features. Subsequently, employing the PHESANT tool [5], we conducted an automated phenome-wide scan within the UK Biobank, exploring the relationships between PRS scores and a wide array of phenotypes. Our investigation encompassed 2,248 phenotypes (Supplementary Table S3), which were classified into five primary groups within the UK Biobank for our analysis: biochemical markers, cognition and mental health, disease diagnoses, health and medical history, and socio-demographics. Adjustments were applied for sex (not included in sex-specific analyses), age, genotype measurement batch, and the first ten principal components to account for any remaining stratification. FDR adjustment was applied to correct P-values for multiple testing.

*Mendelian Randomization (MR) Analysis of blood biomarkers*

Two-sample MR analysis was conducted to examine the potential causal relationship between AD neuropathology traits and PheWAS significant blood assay traits in UK biobank. As exposure variables we considered SNPs with p< 10^−5^ in the AD neuropathological features GWAS summary statistics (ncAD, Braak stage, NP stage). LD-independent SNPs were identified with the clump function in PLINK with r2 threshold of 0.001 and a window size of 1 Mb using 1000 Genomes European samples as reference. The next analyses were performed with the TwosampleMR R package [6]. Inverse-variance weighted (IVW) regression with a multiplicative random effects model was conducted as the primary causal inference. To assess the robustness of our interpretations, we compared the IVW results with two alternative methods that are more resilient to horizontal pleiotropy. Specifically, we employed weighted median MR, which tolerates up to 50% of the instrumental variables being invalid, and MR-Egger regression, which relaxes the assumption that the variants' effects on the outcome are solely mediated through the exposure. Finally, we assessed heterogeneity among the individual SNPs included in the genetic instrument using Cochran’s Q test. If the heterogeneity exists, we applied a multiplicative random-effects model to supplement the results. To determine if any single SNP was disproportionately influencing the effect estimates, we performed leave-one-SNP-out MR analyses. To validate the MR results, we conducted independent analysis using outcomes datasets that excluded UK Biobank participants [7–10]. The MR analysis was performed using the same parameters and thresholds for instrument selection as previously described to test the significance of the associations in an independent analysis. Details of the outcomes for UKB and non-UKB GWASs are provided in Supplementary Table 4.

# Supplementary Results

***Phenome-wide association analysis (PheWAS) and PheWAS-based on MR***

Neuropathology-confirmed AD (ncAD) PRS was associated significantly with the highest number of items (n=36) across the three tested phenotypes, followed by Braak stage PRS (n=10), while we did not observe any significant associations with NP score PRS. In the cognitive function category, we observed significant negative associations between ncAD PRS and “symbol digit substitution: number of correct matches” (beta: -0.0129, p-value: 3.49×10^-5^) and “number of attempted matches” (beta: -0.0126, p-value: 4.22×10^-5^). A positive association was observed with “duration to enter value” (beta: 0.0119, p-value: 1.02×10^-4^). In the family history category, ncAD PRS was positively associated with “siblings: Alzheimer's disease/dementia” (0.011, p-value: 2.52×10^-5)^, “mother with AD/dementia” (beta: 0.095, p-value: 3.49×10-^48^) and “father with AD/dementia” (beta: 0.078, p-value: 2.84×10^-19^).  Within the biomarker category, there were strong positive associations with apolipoprotein B (beta: 0.0365, p-value: 2.91×10^-93^), LDL cholesterol (beta: 0.0269, p-value: 9.28×10^-52^), and Cholesterol (beta: 0.024, p-value: 4.93×10^-39^). Negative associations were observed with C-reactive protein (beta: -0.0339, p-value: 8.16×10^-82^), apolipoprotein A (beta: -0.0106, p-value: 5.42×10^-10^), alanine aminotransferase (beta: -0.0101, p-value: 1.70×10^-9^), and alkaline phosphatase (beta: -0.00926, p-value: 1.12×10^-7^). Additional significant negative associations were found with platelet crit (beta: -0.0087, p-value: 2.22×10^-7^), haematocrit percentage (beta: -0.00708, p-value: 9.90×10^-7), and gamma glutamyltransferase (beta: -0.0056, p-value: 7.97×10^-4^). Significant associations were noted with platelet count (beta: -0.0078, p-value: 4.91×10-6), red blood cell (erythrocyte) count (beta: -0.0065, p-value: 2.22×10^-5^), monocyte count (beta: -0.0064, p-value: 1.77×10^-4^), and HDL cholesterol (beta: -0.0069, p-value: 4.27×10^-5^). Moreover, ncAD PRS was positively associated with dementia-related or broader diagnoses and lipid metabolism disorders but negatively associated with obesity and Obstructive chronic bronchitis. Similarly, Braak stage positive associations with apolipoprotein B (beta: 0.0136, p-value: 2.01×10^-14^), LDL cholesterol (beta: 0.00946, p-value: 9.85×10^-8^), and Cholesterol (beta: 0.0083, p-value: 4.57×10^-6^). Negative associations were observed with C-reactive protein (beta: -0.013, p-value: 3.32×10^-14^), apolipoprotein A (beta: -0.0065, p-value: 1.36×10^-4^). More details can be found in supplementary table 6.

In female-specific PheWAS, ncAD PRS was associated with eight items, Braak PRS with one item and NP stage with one item, while in male-specific PheWAS, ncAD PRS was associated with one item, Braak PRS with two items and NP stage with one item. The majority of the items belonged to the blood assays category. Seven blood assay outcomes were significantly associated with sex-specific PRS, including six for females and one for males. For female ncAD PRS, negative associations were found with white blood cell count (beta: −0.011, p-value: 7.38 × 10^−6^), neutrophil count (beta: −0.009, p-value: 1.35 × 10^−4^), haematocrit percentage (beta: −0.010, p-value: 3.18 × 10^−5^), haemoglobin concentration (beta: −0.009, p-value: 1.21 × 10^−4^), platelet crit (beta: −0.010, p-value: 3.18 × 10^−5^) and platelet count (beta: −0.009, p-value: 1.57× 10^−4^). For male NP stage PRS a positive association was observed with apolipoprotein B (beta: 0.015, p-value: 4.05 × 10^−9^). Among sociodemographic outcomes, one phenotype was significantly negatively associated with PRS for all female neuropathological features: “Place of birth in the UK - north coordinate” (beta: −0.009, p-value: 4.16 × 10^−5^   ncAD female PRS; beta: −0.015, p-value: 1.29 × 10^-10^   Braak female PRS; beta: −0.010, p-value: 1.06 × 10^−5^   NP female PRS). Within the disease diagnoses category, female ncAD PRS was positively associated with celiac disease (beta: 0.102, p-value: 1.53× 10^−4^), and male ncAD and Braak stage PRSs were positively associated with Alzheimer’s disease (beta: 0.149, p-value: 1.80 × 10^−5^ and beta: 0.159, p-value: 5.63 × 10^−6^ respectively). More details can be found in supplementary table 6.

We identified a causal relationship where neuropathology, particularly in case-control GWAS, leads to changes in blood assay traits. Specifically, there was a positive causal association between ncAD and lipid metabolism markers, including cholesterol (beta = 0.082, p = 0.019) and LDL direct (beta = 0.095, p = 0.02). apolipoprotein B (beta = 0.126, p = 0.019) also showed a positive causal link. In contrast, several negative causal relationships were observed. ncAD causally linked to decreased levels of C-reactive protein (CRP) levels (beta = -0.192, p = 0.003), and alkaline phosphatase (beta = -0.03, p = 0.015), alanine aminotransferase (beta = -0.024, p = 0.041), HDL (beta = -0.028, p = 0.043) and apolipoprotein A (beta = -0.068, p = 0.012). Moreover, red blood cell distribution width (beta = -0.037, p = 0.004) and red blood cell count (beta = -0.014, p = 0.007), Platelet count (beta = -0.02, p = 0.014) were also negatively associated with neuropathological changes. Cholesterol, CRP, Platelet crit, LDL direct, and red cell count have been successfully replicated, showing consistent results.

For Braak stage GWAS, the findings indicated a strong causal relationship with increase cholesterol levels (beta = 0.007, p = 0.048) and LDL direct (beta = 0.126, p = 0.003). However, Braak stage was also causally linked to decreased apolipoprotein A levels (beta = -0.061, p = 0.012) and C-reactive protein (beta = -0.176, p = 0.002). The results for Apolipoprotein A and LDL direct have been replicated in an independent study, showing the same direction of effect with significant findings. For reverse direction, we found that LDL direct have positive causal effect on Braak stage (beta = 0.170, p = 0.007) however the result cannot be replicated.

**
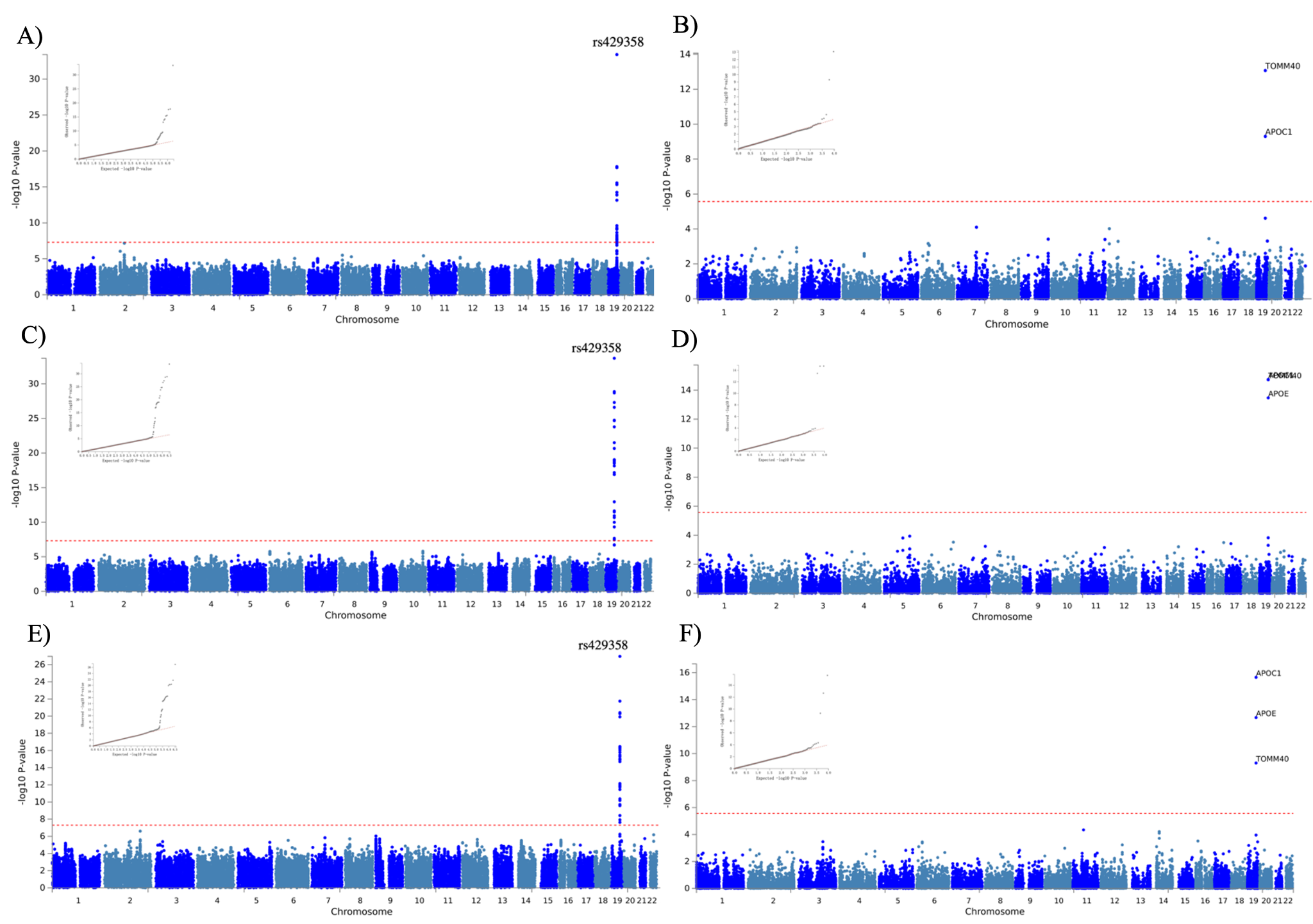
**

# Figure S1: Manhattan and QQ plots of SNP-based and Gene-Based genome-wide association results of neuropathological features of AD in males (n=2366).

Dotted red lines represent the threshold for genome-wide significance (P <5x10^−8^) and Bonferroni correction for the gene-based analyses. A) Neuropathologically-confirmed AD case-control GWAS in males. B) Gene-based analysis for neuropathologically-confirmed AD case-control male-specific sample. C) Braak stage male-specific GWAS. D) Gene-based analysis for Braak stage in maless E) NP score male-specific GWAS. F) Gene-based analysis for NP score in males.


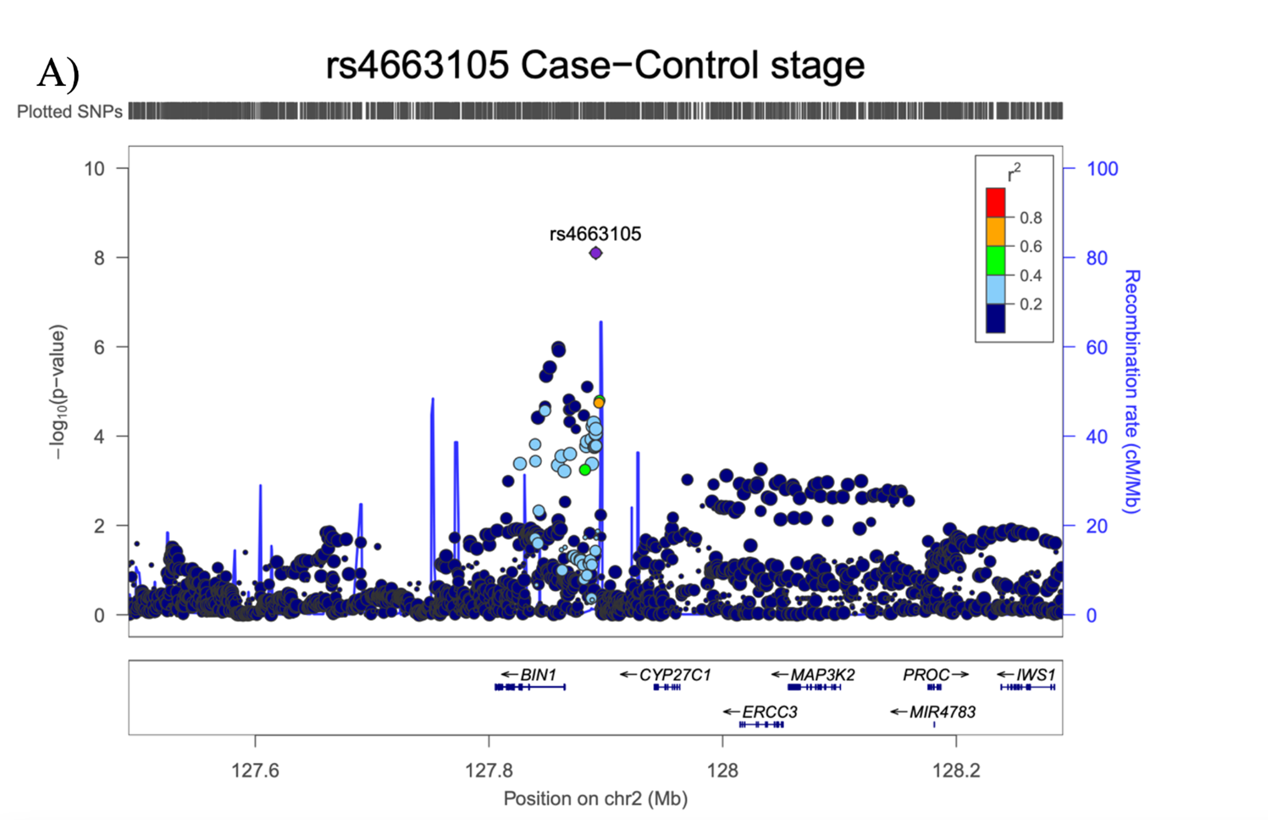


# Figure S2 Regional association plots for locus significantly associated with neuropathological features of AD.

The regional association plot was created using LocusZoom. A) Regional association plots for locus significantly associated with neuropathology-confirmed case-control AD on chromosome 2q14 (BIN1).

| A |
| --- |
| 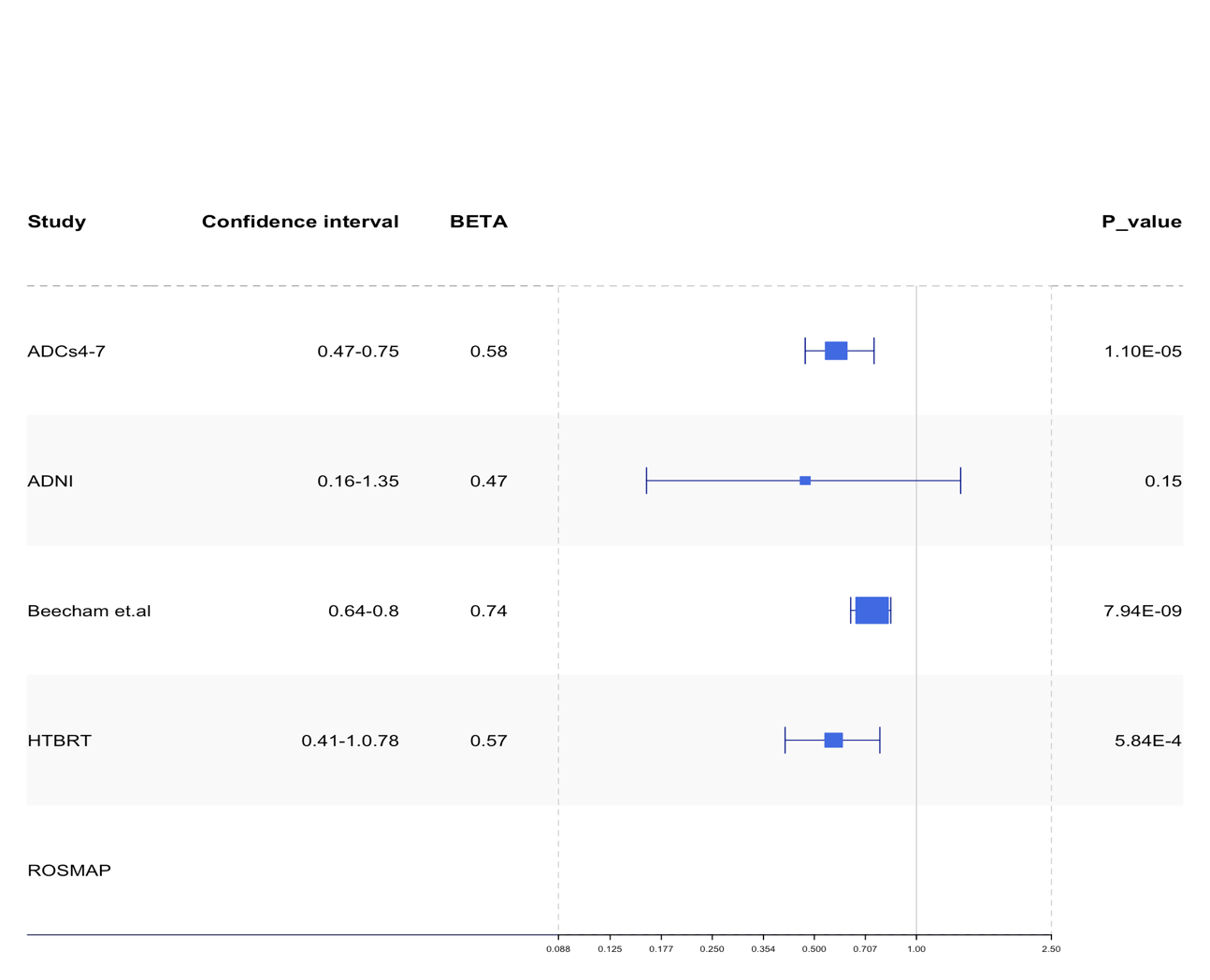 |
| B |
| 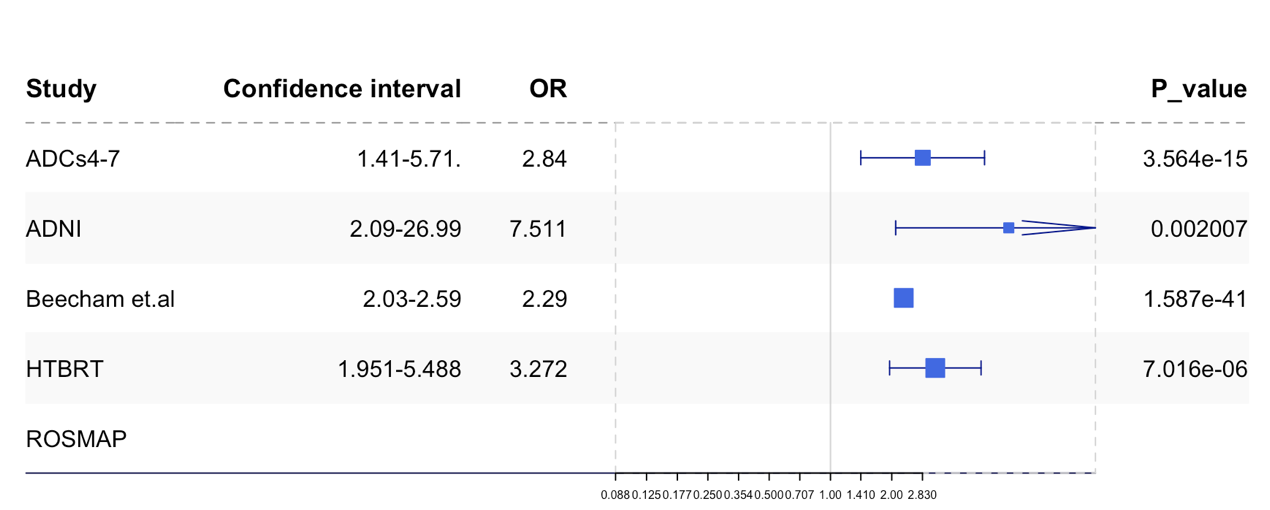 |
| C |
| 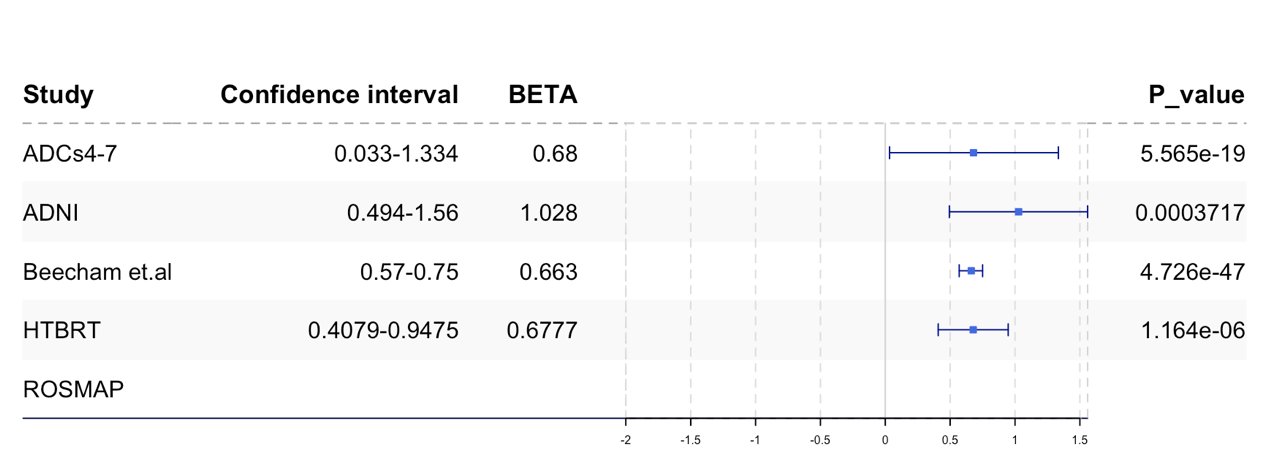 |
| D |
| 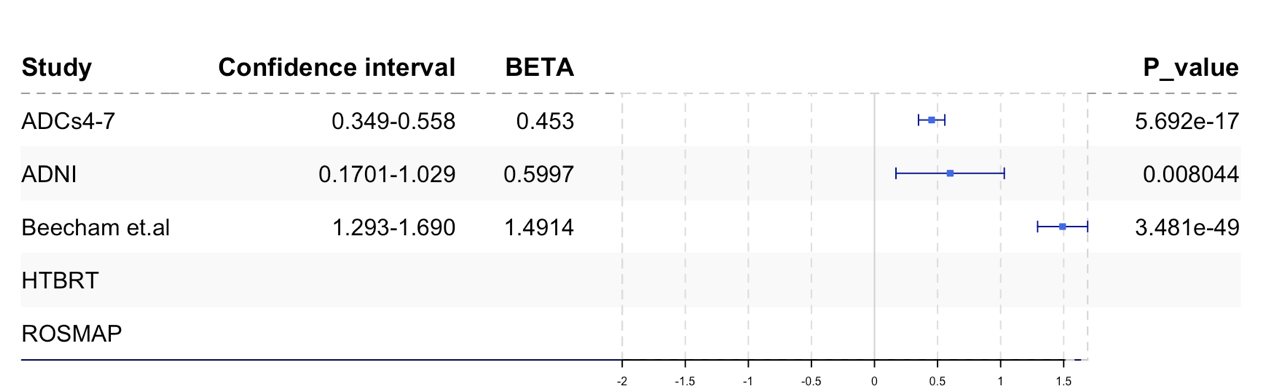 |
|  |

# Figure S3 Forest plots for locus significantly associated with neuropathological features of AD.

The regional association plot was created using R forestplot package. A) Forest plot for significant SNP rs4663105 in neuropathology-confirmed case-control AD GWAS. B) Forest plot for significant SNP rs59007384 in ncAD GWAS. C) Forest plot for significant SNP rs6857 in Braak stage GWAS. E) Forest plot for significant SNP rs11556505 in NP score GWAS.

| A | B |
| --- | --- |
| 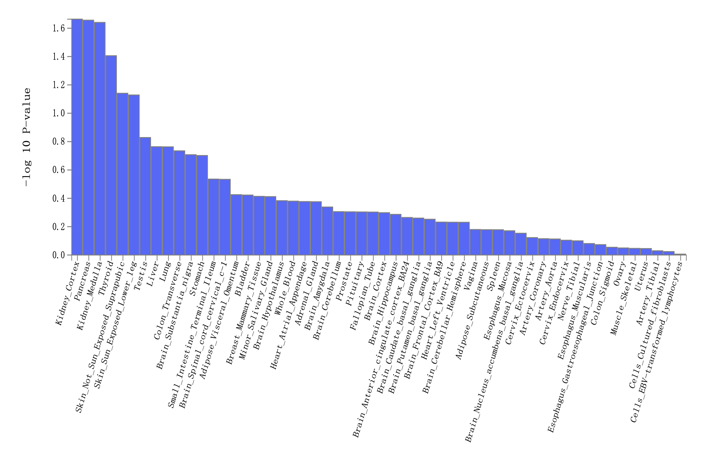 | 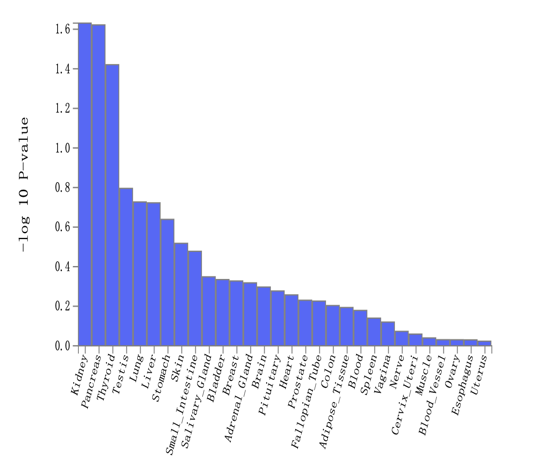 |
| C | D |
| 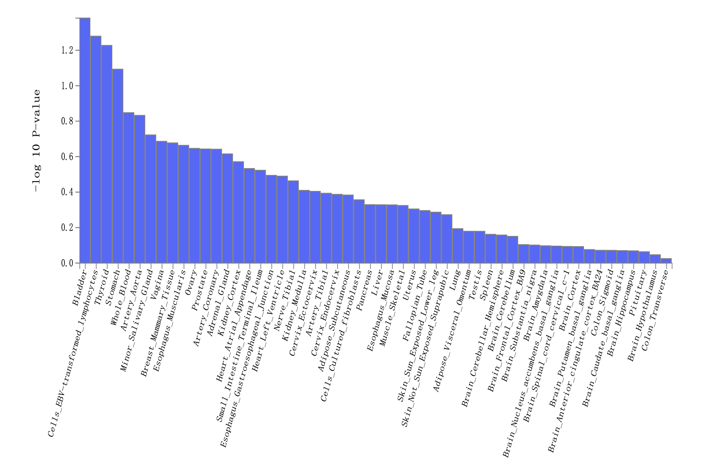 | 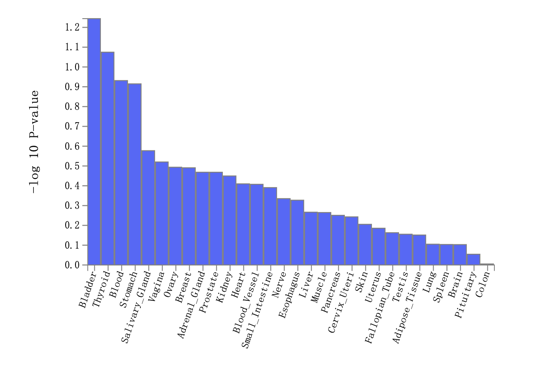 |
| E | F |
| 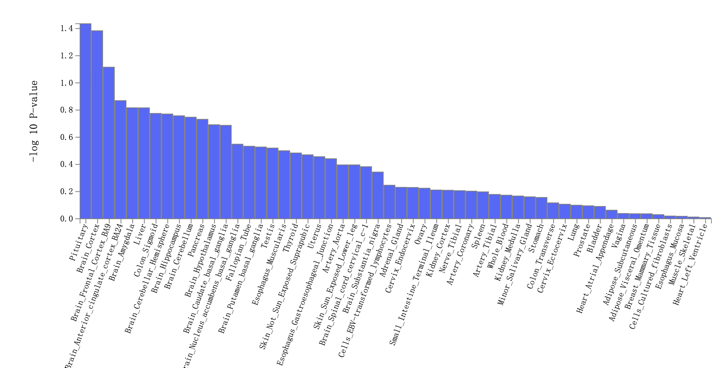 | 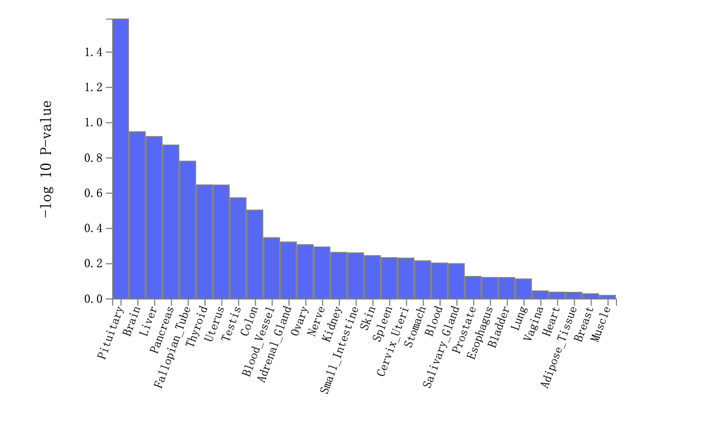 |

# Figure S4: Tissue enrichment analysis for neuropathologically confirmed AD GWAS results.

The analysis was performed in MAGMA using GTEx v8 RNA-seq data 54 and 30 general tissue types. With red are shown the significant results after multiple testing corrections. A) MAGMA ncAD tissue expression analysis for using gene expression per tissue based on GTEx RNA-seq data for 54 specific tissue types. B) MAGMA ncAD tissue expression analysis using gene expression per tissue based on GTEx RNA-seq data for 30 specific tissue types C) MAGMA Braak stage tissue expression analysis for using gene expression per tissue based on GTEx RNA-seq data for 54 specific tissue types. D) MAGMA Braak stage tissue expression analysis using gene expression per tissue based on GTEx RNA-seq data for 30 specific tissue types. E) MAGMA NP score tissue expression analysis for using gene expression per tissue based on GTEx RNA-seq data for 54 specific tissue types. F) MAGMA NP score tissue expression analysis using gene expression per tissue based on GTEx RNA-seq data for 30 specific tissue types.


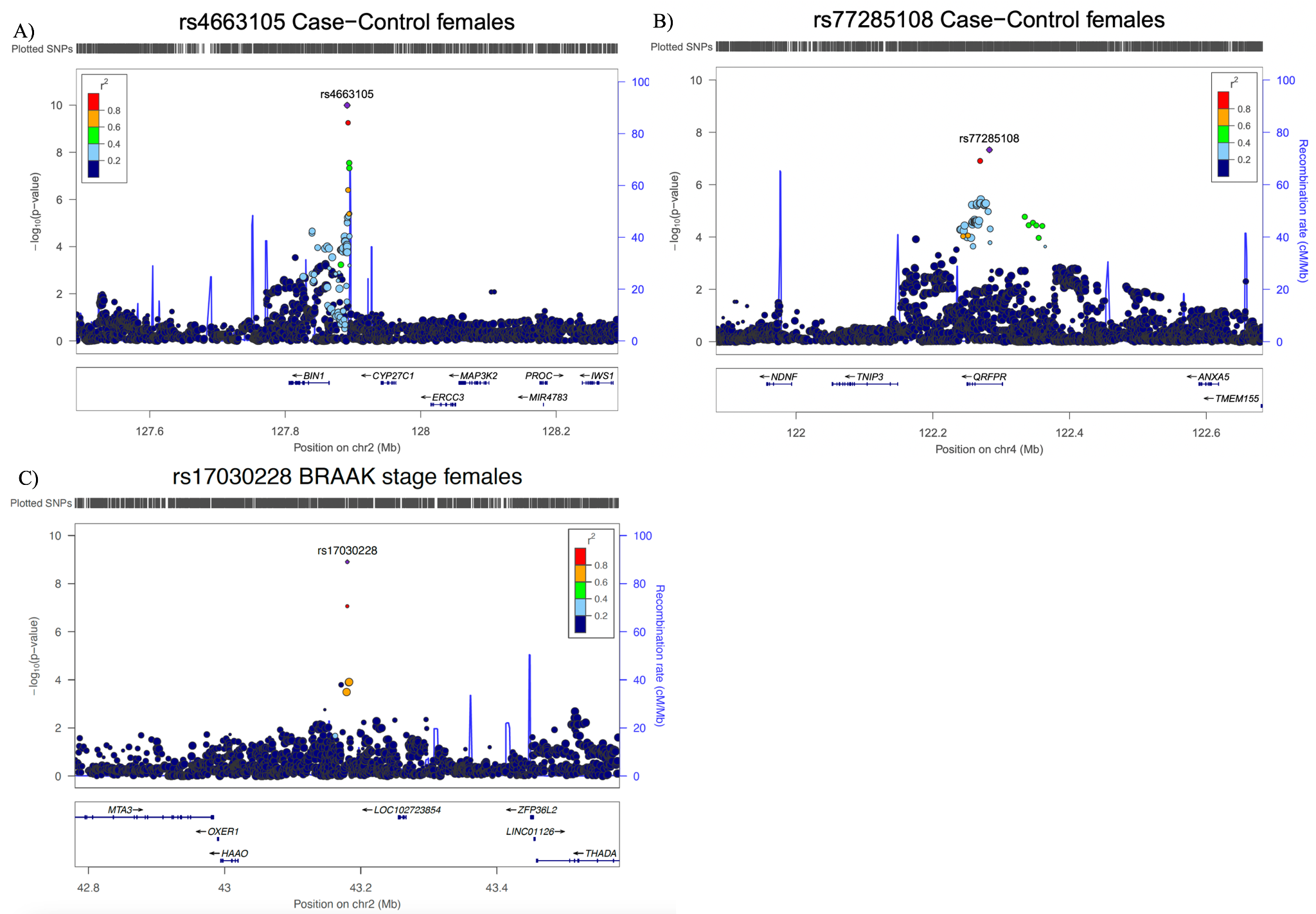


# Figure S5 Regional association plots for locus significantly associated with neuropathological features of AD in females.

The regional association plot was created using LocusZoom. A) Regional association plots for locus significantly associated with neuropathology-confirmed case-control AD in females on chromosome 2q14 (BIN1). B) Regional association plots for locus significantly associated with neuropathology-confirmed case-control AD in females on chromosome 4q27 (QRFPR). C) Regional association plots for locus significantly associated with Braak stage in females on chromosome 2q (AC016735.1/lncRNAs).

| A |
| --- |
| 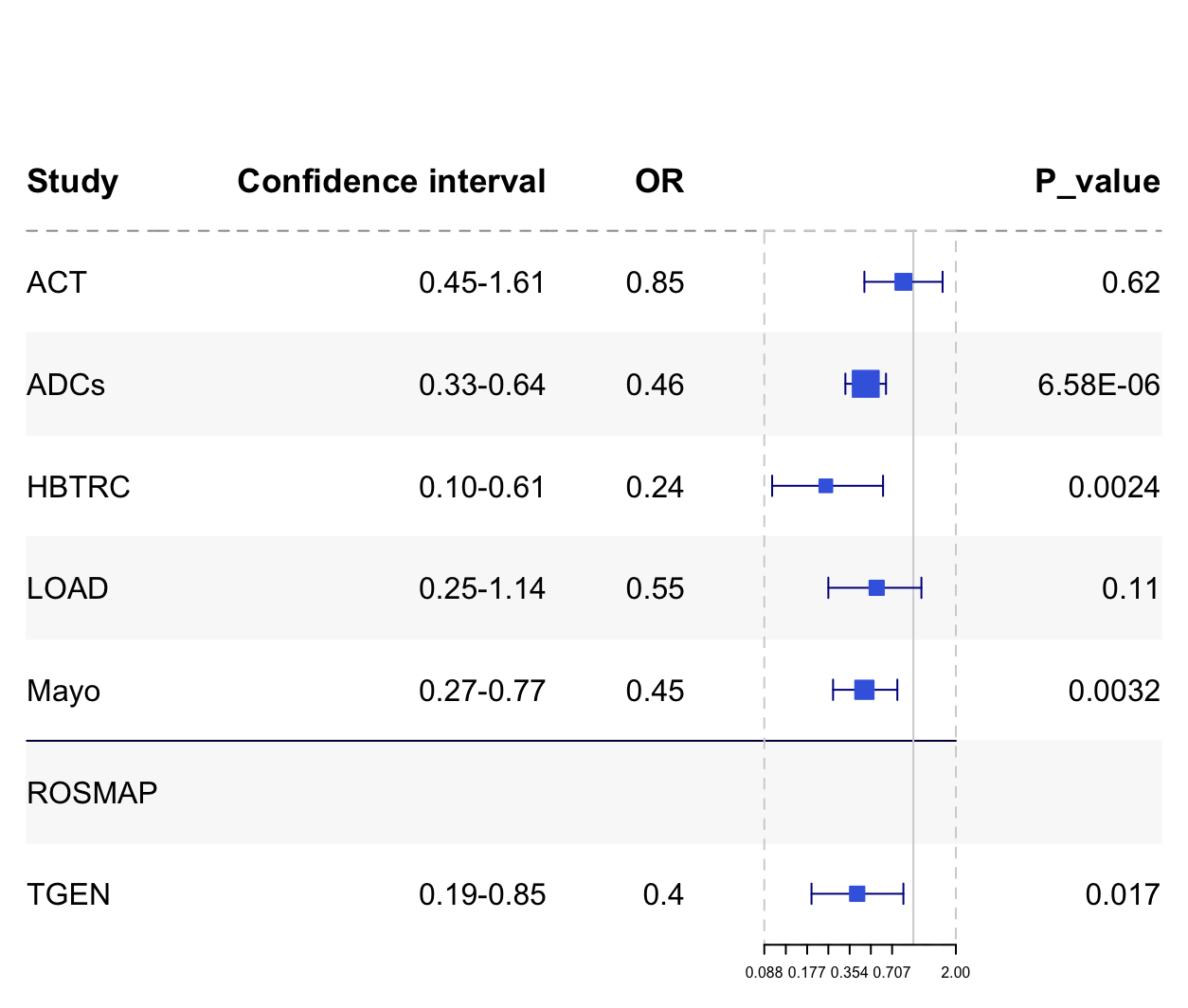 |
| B |
| 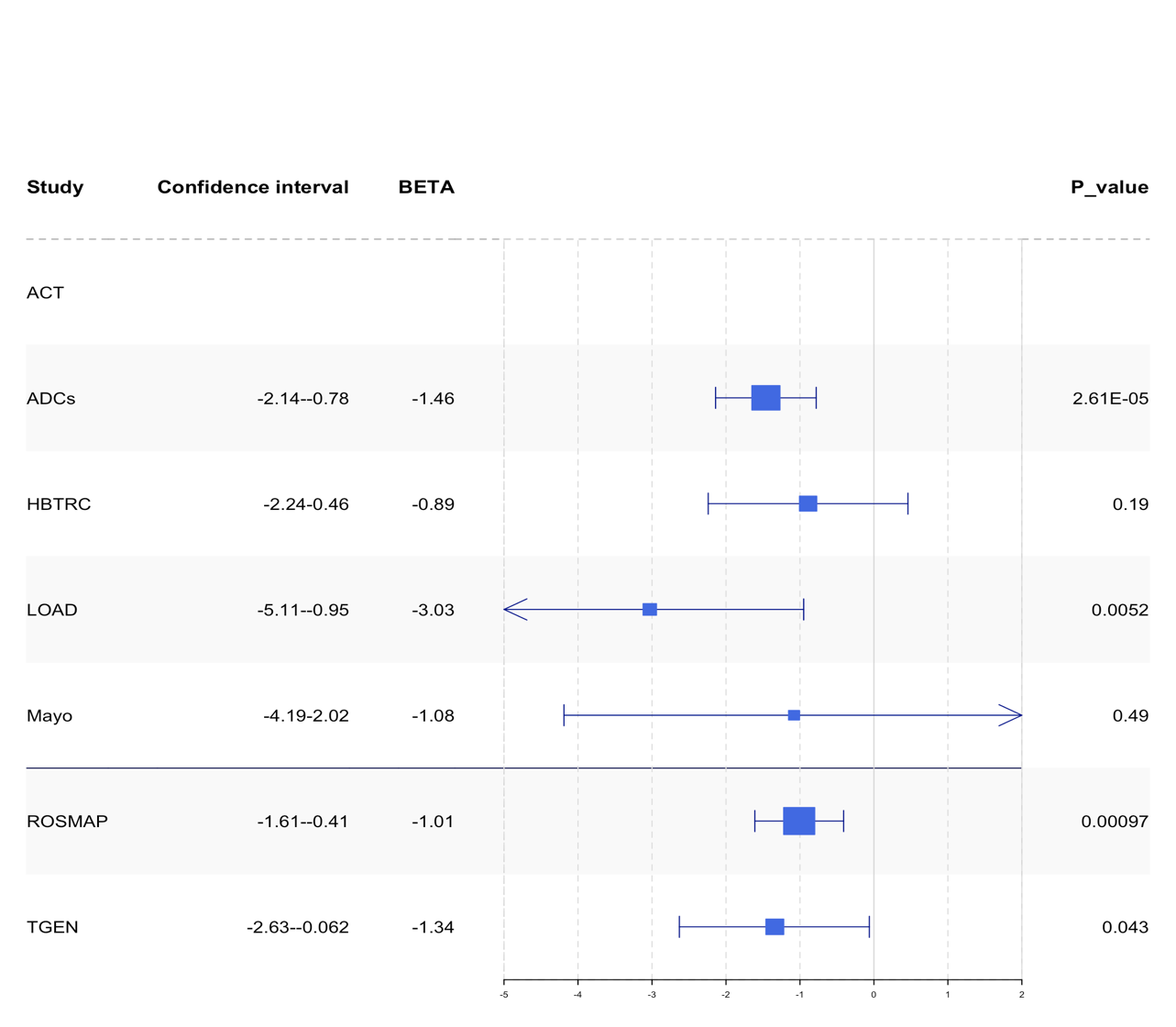 |
| C |
| 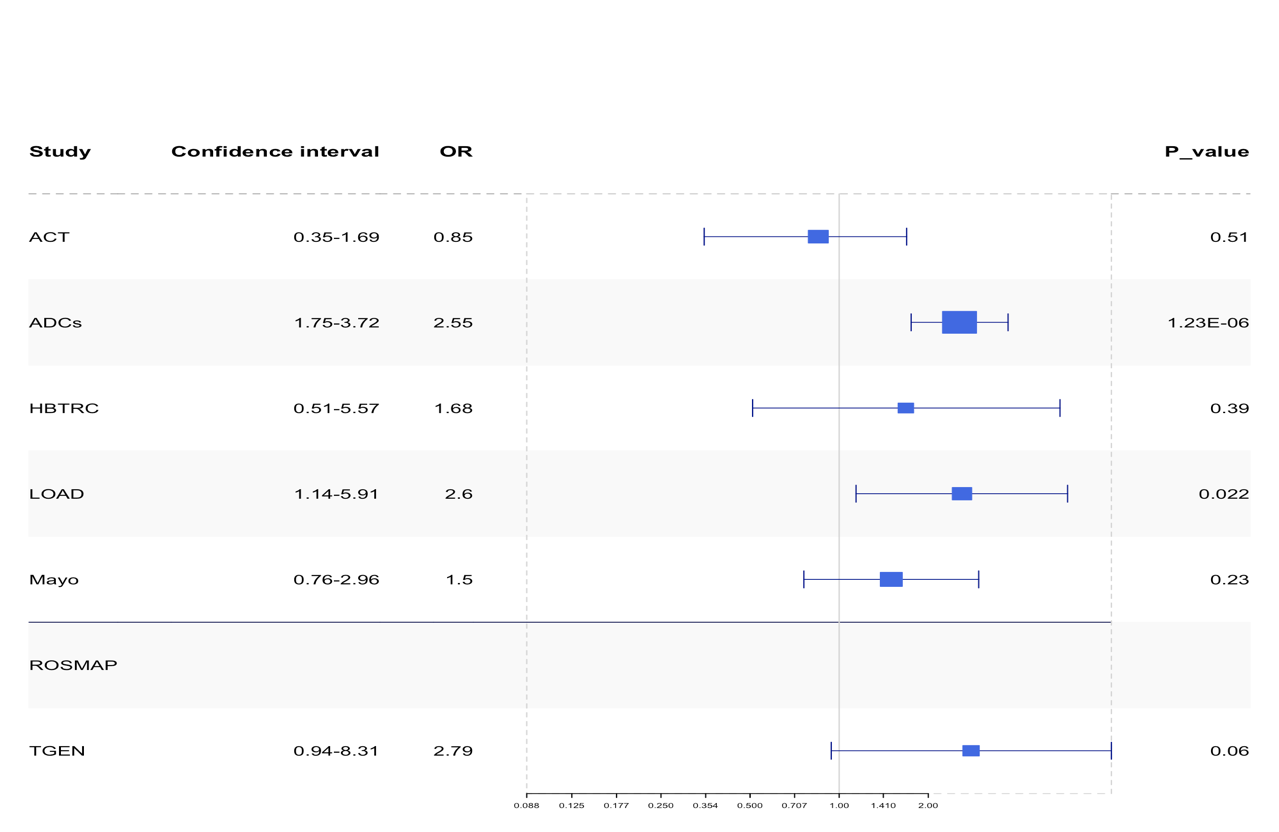 |
| D |
| 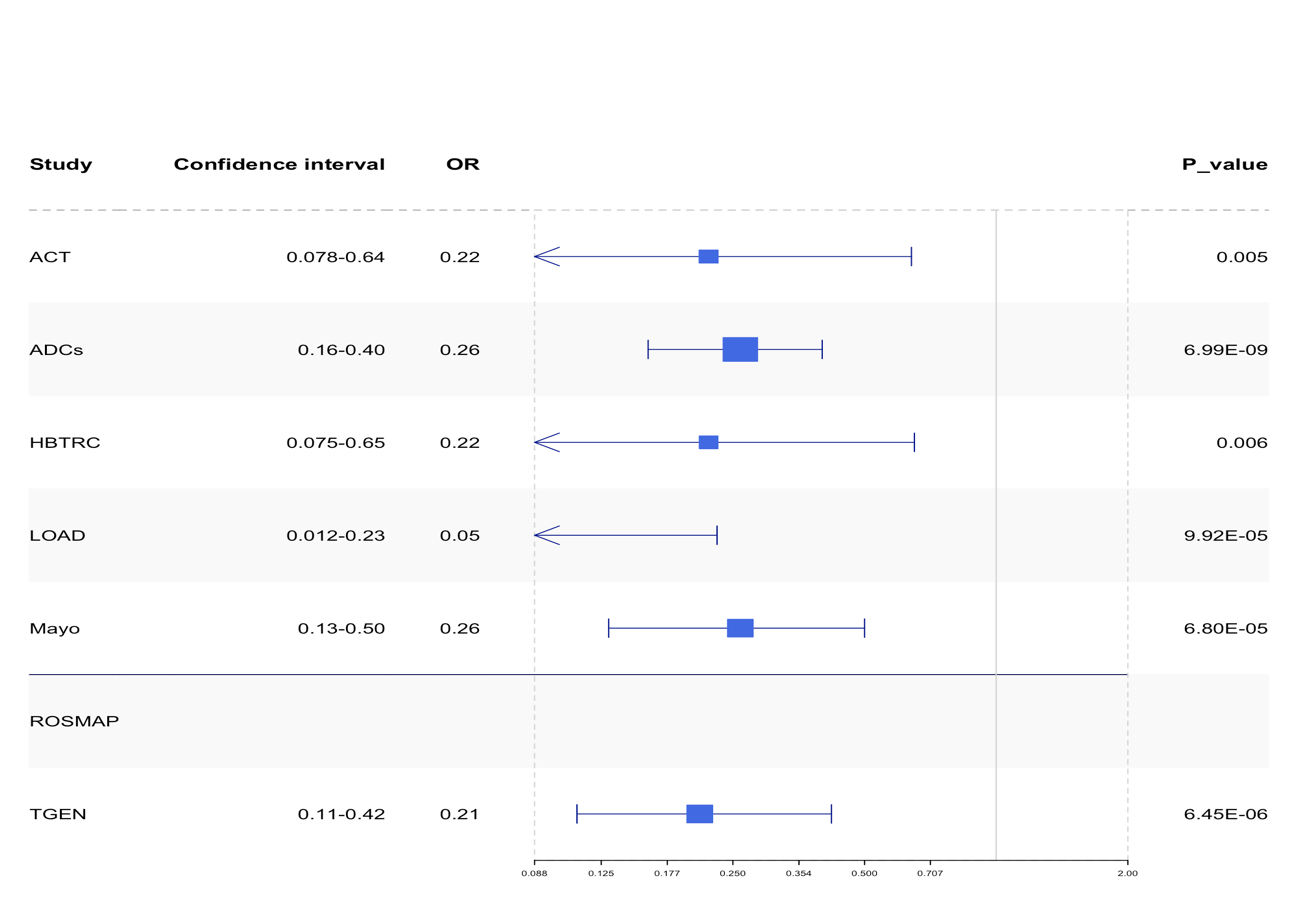 |
| E |
| 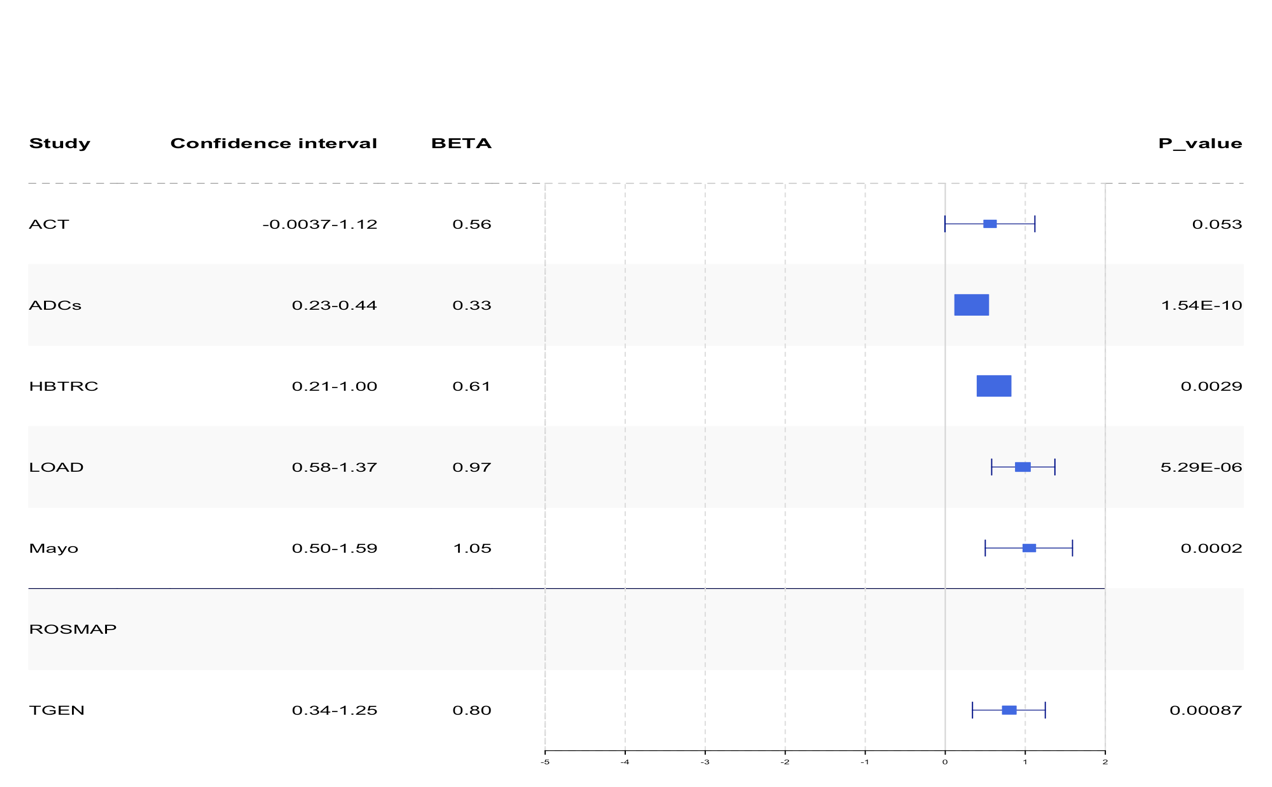 |
| F |
| 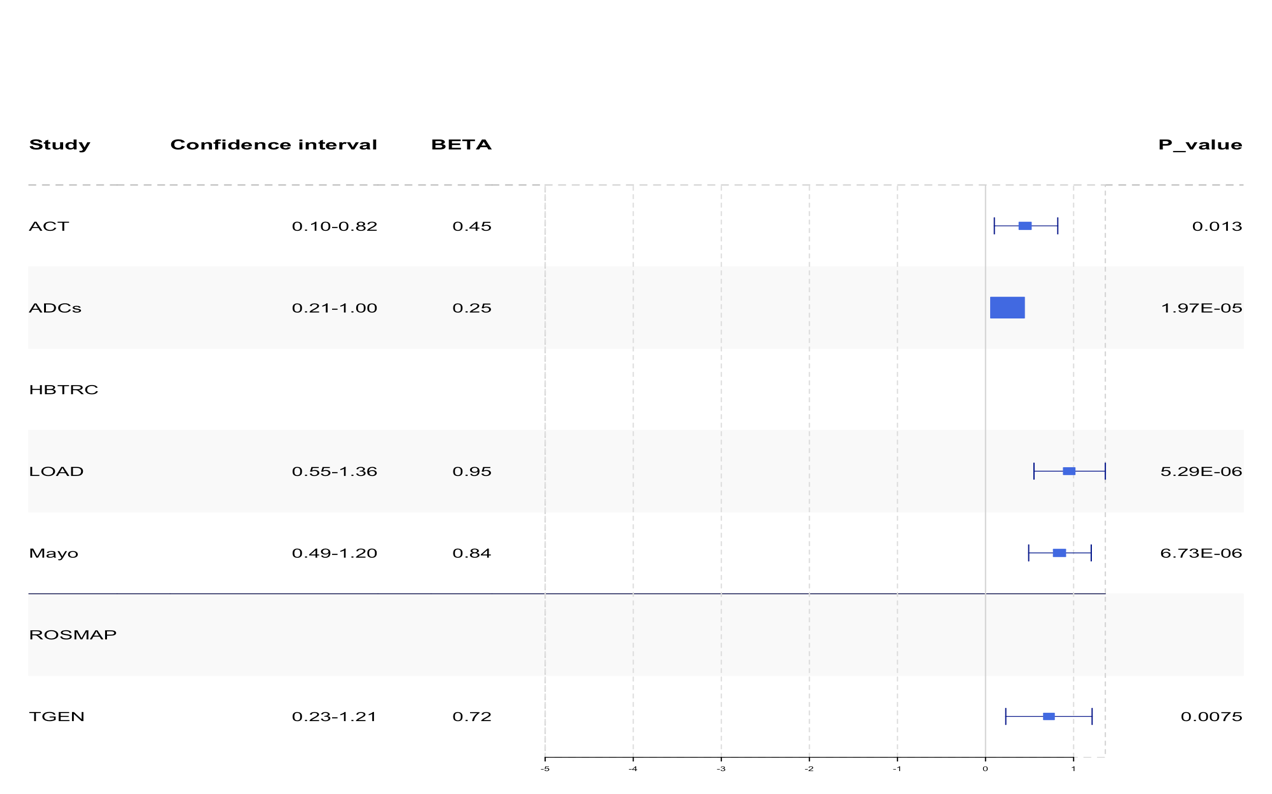 |
| G |
| 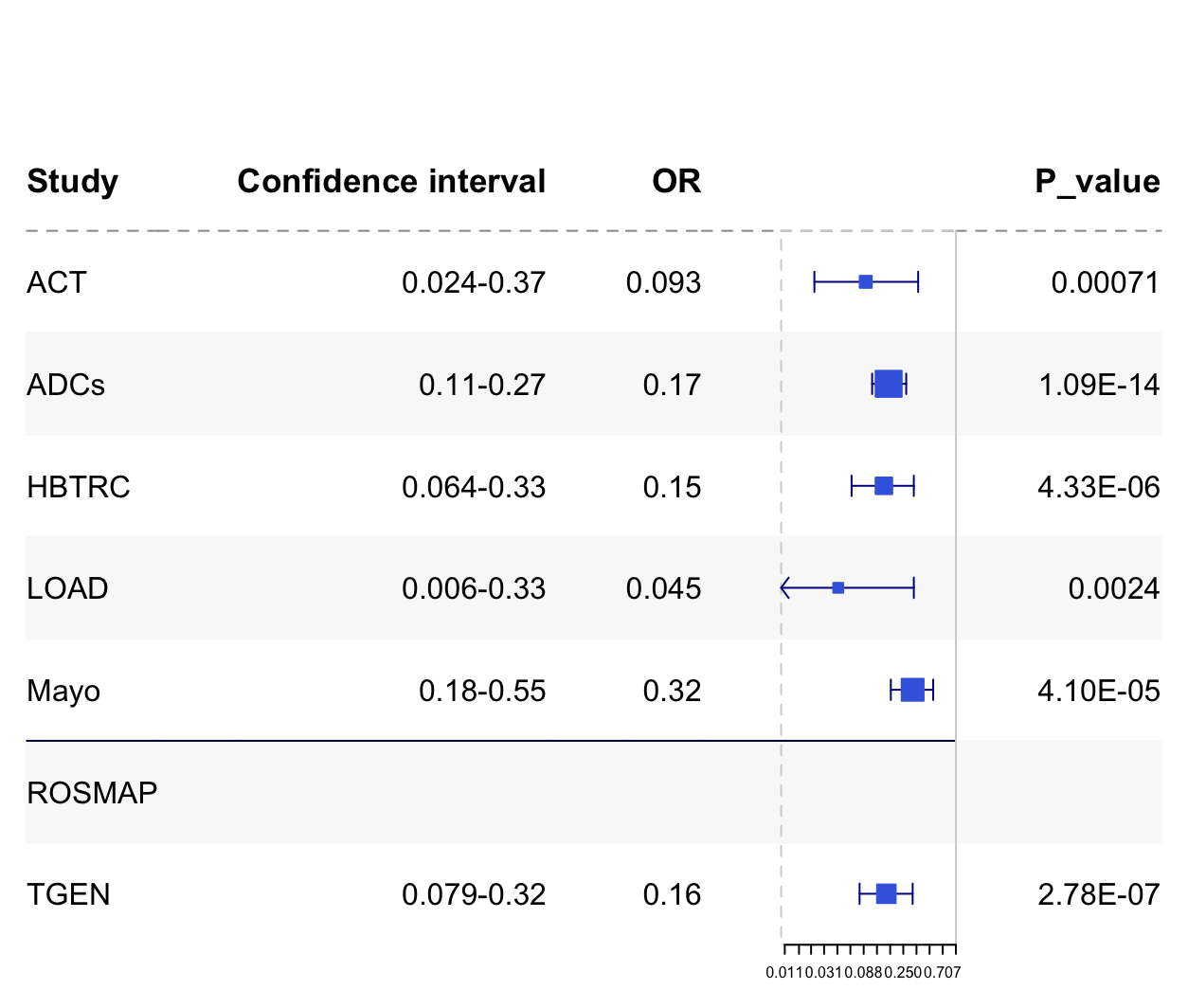 |
| H |
| 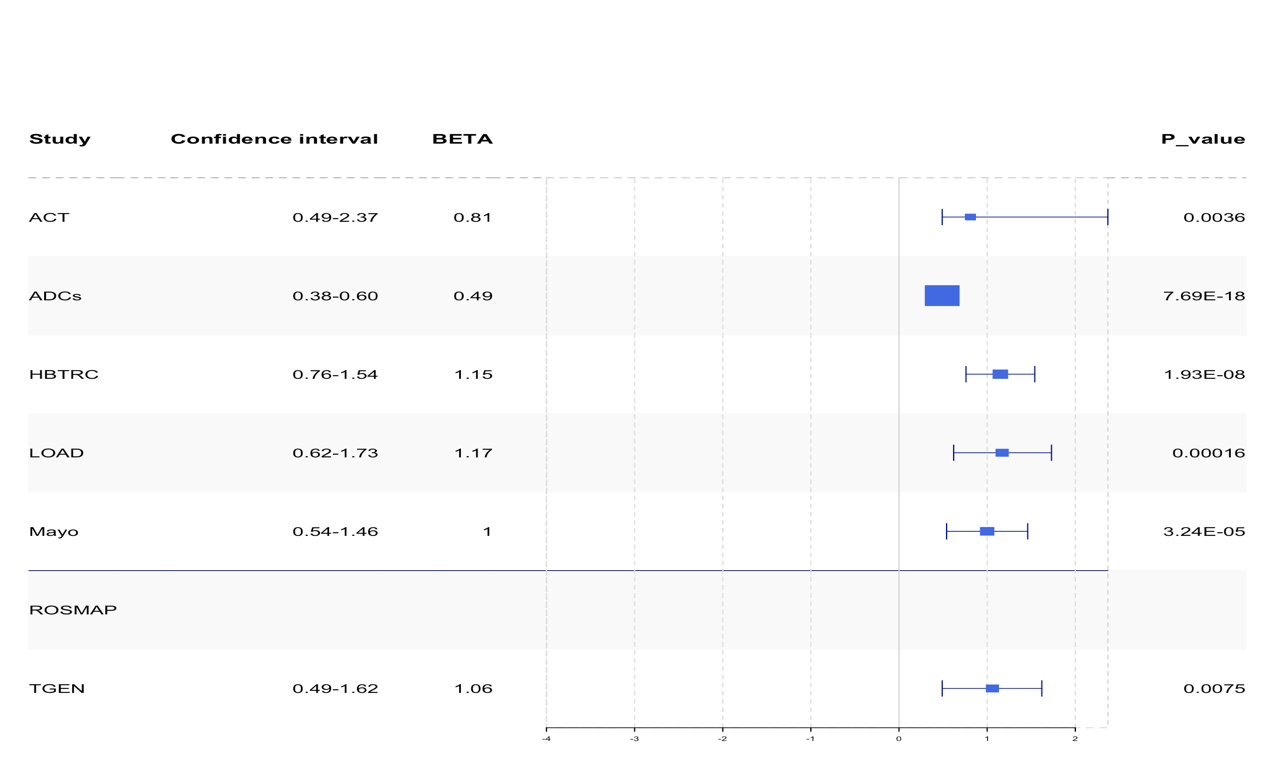 |
| I |
| 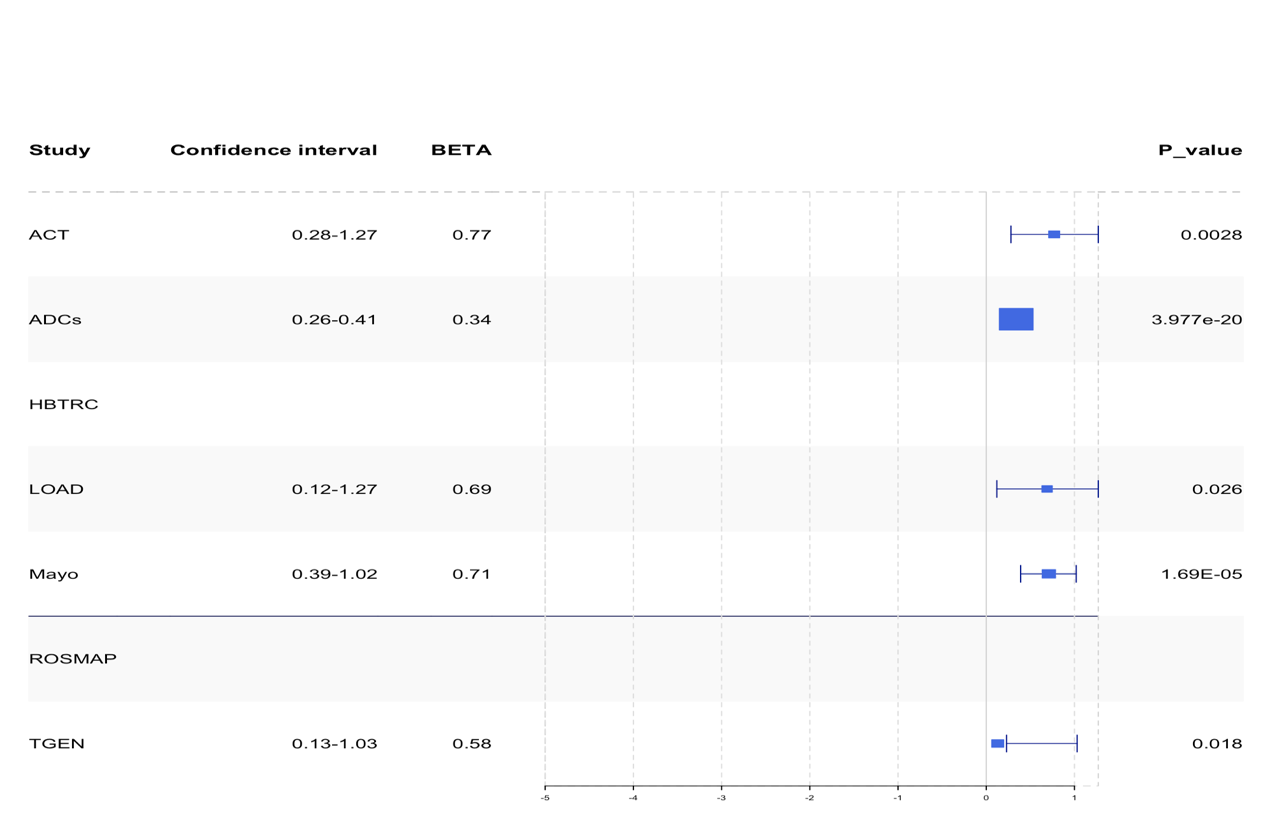 |
| Figure S6 Forest plots for locus significantly associated with sex specific neuropathological features of AD. The regional association plot was created using R forestplot package. A) Forest plot for significant SNP rs4663105 in neuropathology-confirmed case-control AD GWAS for female. B) Forest plot for significant SNP rs17030228 in Braak stage GWAS for female. C) Forest plot for significant SNP rs77285108 in neuropathology-confirmed case-control AD GWAS for female. D) Forest plot for significant SNP rs429358 in neuropathology-confirmed case-control AD GWAS for female. E) Forest plot for significant SNP rs429358 in Braak stage GWAS for female. F) Forest plot for significant SNP rs429358 in NP score GWAS for female. G) Forest plot for significant SNP rs429358 in neuropathology-confirmed case-control AD GWAS for male. EH Forest plot for significant SNP rs429358 in Braak stage GWAS for male. I) Forest plot for significant SNP rs429358 in NP score GWAS for male. |

| A | B |
| --- | --- |
| 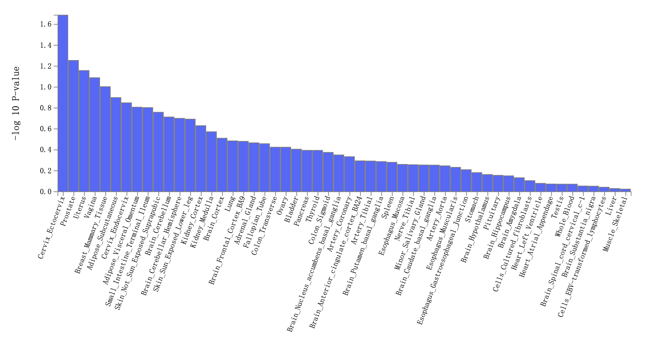 | 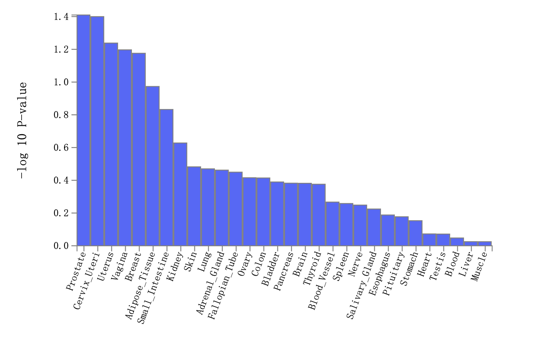 |
| C | D |
| 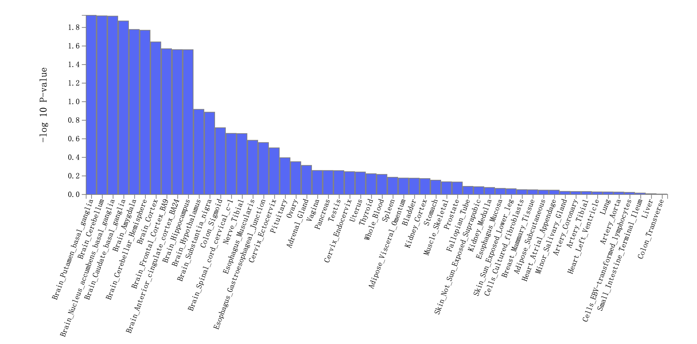 | 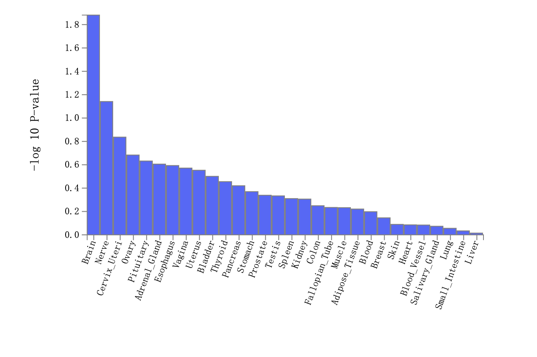 |
| E | F |
| 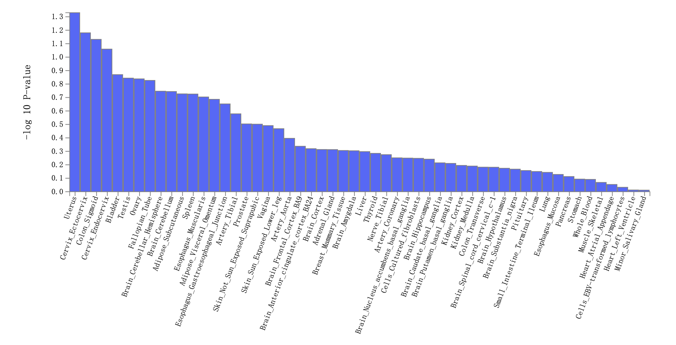 | 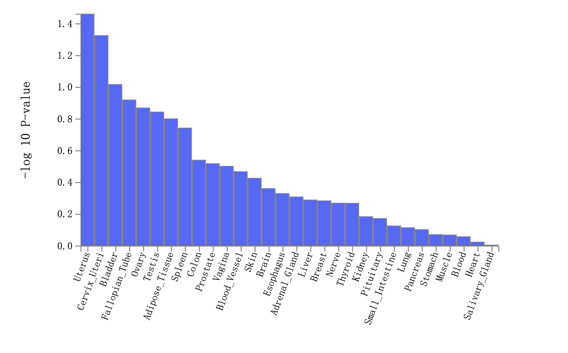 |
| G | H |
| 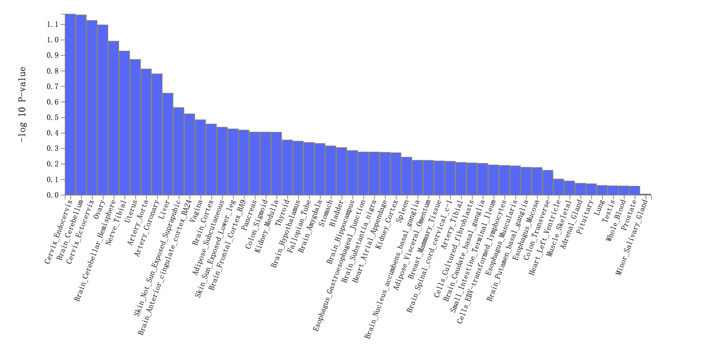 | 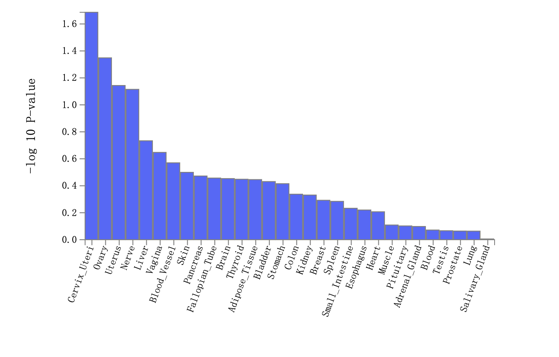 |
| I | J |
| 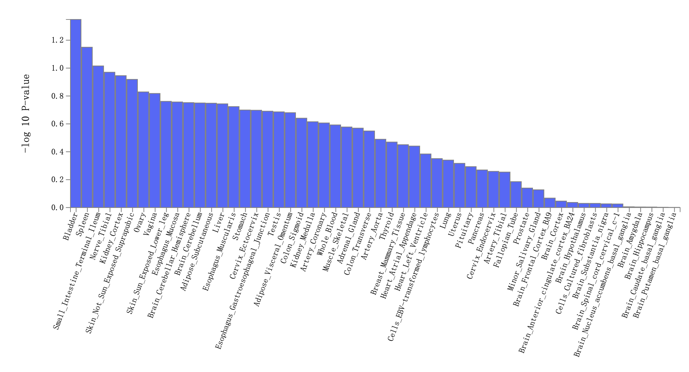 | 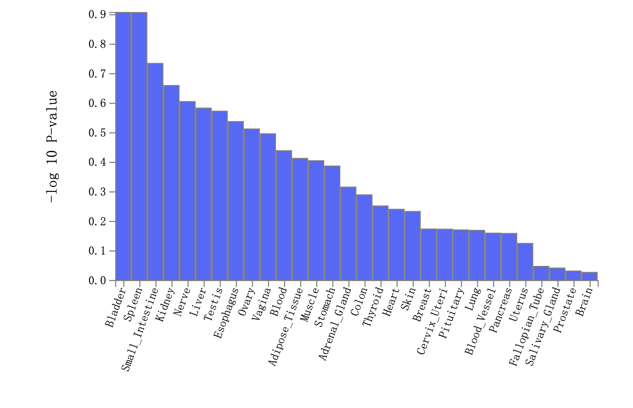 |

# Figure S7: Tissue enrichment analysis for neuropathologically confirmed AD sex specific GWAS results.

The analysis was performed in MAGMA using GTEx v8 RNA-seq data 54 and 30 general tissue types. With red are shown the significant results after multiple testing corrections. A) MAGMA ncAD male-specific tissue expression analysis for using gene expression per tissue based on GTEx RNA-seq data for 54 specific tissue types. B) MAGMA ncAD male-specific tissue expression analysis using gene expression per tissue based on GTEx RNA-seq data for 30 specific tissue types C) MAGMA Braak stage male-specific tissue expression analysis for using gene expression per tissue based on GTEx RNA-seq data for 54 specific tissue types. D) MAGMA Braak stage male-specific tissue expression analysis using gene expression per tissue based on GTEx RNA-seq data for 30 specific tissue types. E) MAGMA NP score male-specific tissue expression analysis for using gene expression per tissue based on GTEx RNA-seq data for 54 specific tissue types. F) MAGMA NP score male-specific tissue expression analysis using gene expression per tissue based on GTEx RNA-seq data for 30 specific tissue types. G) MAGMA ncAD female-specific tissue expression analysis for using gene expression per tissue based on GTEx RNA-seq data for 54 specific tissue types. H) MAGMA ncAD female-specific tissue expression analysis using gene expression per tissue based on GTEx RNA-seq data for 30 specific tissue types. I) MAGMA Braak stage female-specific tissue expression analysis for using gene expression per tissue based on GTEx RNA-seq data for 54 specific tissue types. J) MAGMA Braak stage female-specific tissue expression analysis using gene expression per tissue based on GTEx RNA-seq data for 30 specific tissue types

.

# Reference

1. Fillenbaum GG, van Belle G, Morris JC, Mohs RC, Mirra SS, Davis PC, et al. Consortium to Establish a Registry for Alzheimer’s Disease (CERAD): The first twenty years. Alzheimer’s Dement. 2008.

2. Braak H, Braak E. Neuropathological stageing of Alzheimer-related changes. Acta Neuropathol. 1991.

3. Ge T, Chen CY, Ni Y, Feng YCA, Smoller JW. Polygenic prediction via Bayesian regression and continuous shrinkage priors. Nat Commun. 2019;

4. Bycroft C, Freeman C, Petkova D, Band G, Elliott LT, Sharp K, et al. The UK Biobank resource with deep phenotyping and genomic data. Nature. 2018;

5. Millard LAC, Davies NM, Gaunt TR, Smith GD, Tilling K. Software application profile: PHESANT: A tool for performing automated phenome scans in UK Biobank. Int J Epidemiol. 2018;

6. Hemani G, Zheng J, Elsworth B, Wade KH, Haberland V, Baird D, et al. The MR-base platform supports systematic causal inference across the human phenome. Elife. 2018;

7. Sun BB, Maranville JC, Peters JE, Stacey D, Staley JR, Blackshaw J, et al. Genomic atlas of the human plasma proteome. Nature. 2018;

8. Mack S, Coassin S, Rueedi R, Yousri NA, Seppälä I, Gieger C, et al. A genome-wide association meta-analysis on lipoprotein (a) concentrations adjusted for apolipoprotein (a) isoforms. J Lipid Res. 2017;

9. Dennis JK, Sealock JM, Straub P, Lee YH, Hucks D, Actkins KE, et al. Clinical laboratory test-wide association scan of polygenic scores identifies biomarkers of complex disease. Genome Med. 2021;

10. Astle WJ, Elding H, Jiang T, Allen D, Ruklisa D, Mann AL, et al. The Allelic Landscape of Human Blood Cell Trait Variation and Links to Common Complex Disease. Cell. 2016;
